# Effects of Shenling Baizhu powder on intestinal microflora metabolites and liver mitochondrial energy metabolism in nonalcoholic fatty liver mice

**DOI:** 10.3389/fmicb.2023.1147067

**Published:** 2023-07-18

**Authors:** Zheng Yao, Jia Guo, Bing Du, Li Hong, Ying Zhu, Xiaoyi Feng, Yuanlu Hou, Anhua Shi

**Affiliations:** ^1^School of Basic Medical Sciences, Yunnan University of Chinese Medicine, Kunming, China; ^2^The Key Laboratory of Microcosmic Syndrome Differentiation, Education Department of Yunnan, Yunnan University of Chinese Medicine, Kunming, China; ^3^Yunnan Key Laboratory of Integrated Traditional Chinese and Western Medicine for Chronic Disease in Prevention and Treatment, Kunming, China; ^4^Dongtai City Hospital of Traditional Chinese Medicine, Dongtai, China; ^5^Heilongjiang Provincial Hospital of Traditional Chinese Medicine, Harbin, China; ^6^Wuhan Special Service Recuperation Center, Wuhan, China

**Keywords:** Shenling Baizhu powder, NAFLD, intestinal flora, short-chain fatty acid, mitochondria, UCP2, AMPK, IF1

## Abstract

**Background & purpose:**

Non-alcoholic fatty liver disease (NAFLD) is characterised by the excessive accumulation of triglycerides in the liver. Shenling Baizhu powder (SLBZP) is formulated from various natural medicinal plants that protect the liver and are used to treat intestinal diseases. SLBZP improves the symptoms of NAFLD. However, its mechanism of action remains unclear. Herein, we investigated the ameliorative effect of SLBZP on model mice with high-fat-diet (HFD)-induced NAFLD. Additionally, we evaluated the impact of SLBZP on the intestinal flora and its metabolites and mitochondrial energy metabolism in NAFLD.

**Methods:**

We used HFD to establish a mouse model of NAFLD. Different drug interventions were administered. We measured serum biochemical indices. Liver sections were visualised with hematoxylin–eosin and oil red O staining. *16S rDNA* amplicon sequencing technology was used to analyse the diversity and abundance of the intestinal flora. Short-chain fatty acids (SCFAs) in the intestinal contents were detected using GC-MS. Liver tissue was sampled to detect mitochondrial membrane functional indices. Western blotting was used to determine the levels of mitochondrial pathway-related proteins, namely, uncoupling protein 2 (UCP2), adenosine monophosphate-activated protein kinase (AMPK) and inhibitory factor 1 (IF1) of F1Fo ATP synthesis/hydrolase, in the liver.

**Results:**

The spleen-invigorating classic recipe of SLBZP reduced liver lipid deposition in mice with HFD-induced NAFLD. Additionally, SCFAs produced by intestinal flora metabolism regulated the UCP2/AMPK/IF1 signalling pathway involved in liver mitochondrial energy metabolism to improve the liver mitochondrial membrane permeability, respiratory state and oxidative phosphorylation efficiency of mice with NAFLD. Finally, SLBZP increased the liver ATP level.

**Conclusion:**

Our results suggest that the therapeutic effect of SLBZP on NAFLD is related to the regulation of hepatic mitochondrial energy metabolism by intestinal flora and its metabolites and is possibly associated with the UCP2/AMPK/IF1 signalling pathway.

## Graphical Abstract

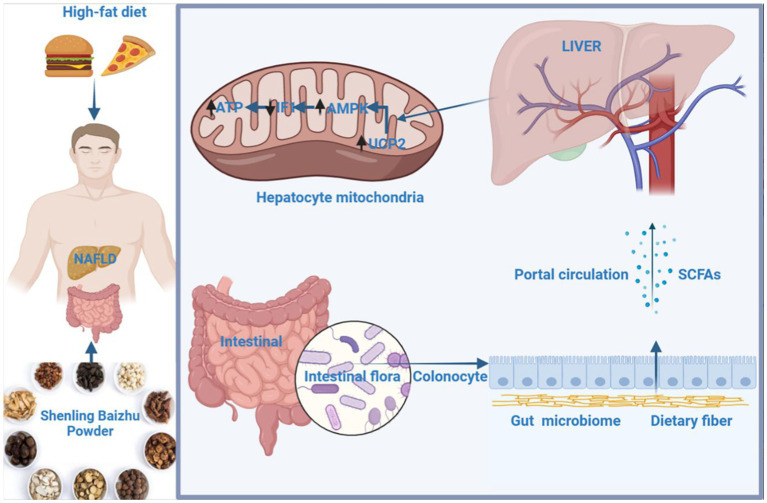

## 1. Introduction

Non-alcoholic fatty liver disease (NAFLD) is a metabolic disease caused by factors other than alcohol and definite liver damage. It is characterised by diffuse hepatocellular steatosis and triglyceride accumulation (Wong et al., 2018). NAFLD involves the progression from simple hepatic steatosis to non-alcoholic steatohepatitis (NASH) through inflammation, hepatocellular injury and progressive fibrosis. It eventually leads to a dynamic state of cirrhosis and hepatocellular carcinoma (Sanyal, 2019). Globally, approximately 25% of individuals suffer from NAFLD (Murag et al., 2021). It affects about 30% of the general population in western countries (Li et al., 2019). It is estimated that the prevalence of NASH in China, France, Germany, Italy, Japan, Spain, the United Kingdom and the United States will increase by 56% between 2016 and 2030 (Estes et al., 2018). However, despite the high incidence and severity of NAFLD, it still lacks an effective treatment strategy (Wang et al., 2011).

The hypothesis of “multiple strikes” has been increasingly recognised as an explanation of how NAFLD forms (Horton et al., 2002; Buzzetti et al., 2016). According to this hypothesis, the intestinal microbiome plays a role in the formation and development of NAFLD through the intestinal–hepatic axis together with insulin resistance and endotoxin (Guarner and Malagelada, 2003). Intestinal flora decomposes unabsorbed substances into substances that the human body can absorb, including short-chain fatty acids (SCFAs).

SCFAs play a key role in adipogenesis (McNeil, 1984), in which acetic, propionic and butyric acids influence cholesterol, free fatty acid and glucose metabolism (den Besten et al., 2013a) to reduce fat accumulation in the body. Conversely, intestinal flora transport decomposed nutrients to the liver through the enterohepatic circulation and simultaneously activate the farnesoid X receptor (Makishima et al., 1999; Parks et al., 1999; Wang et al., 1999), increasing the consumption of excess fat (Savkur et al., 2005). Oxidative stress of the liver mitochondria caused by lipid accumulation is a vital link that leads to the development from a fatty liver to a state of inflammation (Zhou et al., 2020). The normal function of the mitochondrial membrane in the liver is partly reflected in the regulation of membrane permeability by mitochondria, whereas the normal respiratory function of mitochondria is reflected in their ability to produce ATP *via* oxidative phosphorylation (Cortez-Pinto and Machado, 2009). The key proteins of mitochondrial energy metabolism include UCP2, AMPK and IF1. UCP2 is a transmembrane protein located on the inner membrane of mitochondria. UCP2 directly transports protons into the mitochondrial matrix, thus uncoupling oxidative phosphorylation and reducing ATP synthesis (Cortez-Pinto and Machado, 2009). AMPK is an important kinase that regulates energy homeostasis, which can be regulated by adenylate. When the level of ADP in cells increases and that of ATP decreases, ATP synthesis increases by promoting mitochondrial catabolism while reducing unnecessary energy consumption and maintaining the ratio of ADP/ATP (Bullon et al., 2016). AMPK also inhibits the activity of downstream proteins (such as acetyl-CoA carboxylase II, the rate-limiting enzyme of fat oxidation) and accelerates lipid oxidation in hepatocytes (Vancura et al., 2018). IF1 is located in the mitochondrial matrix. When H^+^ concentration increases in the matrix, IF1 inhibits the synthesis of F1Fo ATP and the ability of hydrolase to hydrolyse ATP and prevents energy consumption (Mnatsakanyan and Jonas, 2020). Owing to disordered lipid metabolism and lipid accumulation in liver cells, UCP2 expression significantly increases, as does the proton concentration in the matrix, leading to an increase in IF1 expression. Decreased AMPK level or activity results in the failure to regulate energy homeostasis and inhibit fatty acid oxidation, accelerating the process of NAFLD. Therefore, the respiratory state of mitochondria, the change in membrane potential and the ratio of phosphorus to oxygen can reflect the energy metabolism of mitochondria.

Although basic research on NAFLD is increasingly being performed, a drug for treating NAFLD is still lacking. In this context, it is notable that the curative effects of traditional Chinese medicine (TCM) are being affirmed by clinical practice and research (Hu et al., 2011; Hu, 2015; Zhang et al., 2015). Within TCM, it is considered that NAFLD caused by long-term overeating is characterised by spleen deficiency, stagnation of phlegm, blood stasis and intestinal symptoms such as diarrhoea and loose stool. Modern research has shown that dysregulation of the intestinal microbiome is important in the pathogenesis of various chronic liver diseases, and digestive tract symptoms are consistent with spleen deficiency syndromes in TCM (Yanning, 2017). Therefore, invigorating the spleen has high potential for treating NAFLD. Our previous study showed that early NAFLD can be improved and treated by strengthening the spleen and removing blood stasis (Bian Yao et al., 2014; Yang Yinhua et al., 2015; Zhang et al., 2015; Zhang Shunzhen et al., 2015; Yao et al., 2017).

SLBZP comes from a Taiping Huimin Hejiju prescription that emerged in the Song Dynasty, which is a classic prescription for invigorating the spleen. It is composed of ginseng, yam, coix seed, Poria, Fructus Amomi, white lentil, licorice, Platycodonis Radix, atractylodes and lotus seed (Chen et al., 2018; Fan, 2018) and is widely used in experimental studies and clinical practice. In a previous study, SLBZP was used to treat functional diarrhoea with spleen deficiency in rats (Xiao et al., 2021). A large dose of SLBZP improved functional diarrhoea with spleen deficiency in rats and enhanced the body’s immunity. Another study established a rat colitis model using a colitis inducer [2,4,6-trinitrobenzene sulfonic acid (TNBS)] and investigated the protective effect and mechanism of action of SLBZP on TNBS-induced colitis (Rao et al., 2022). The results revealed that SLBZP improved the symptoms of TNBS-induced colitis in rats and reduced the secretion of proinflammatory cytokines. SLBZP inhibits cell apoptosis and enhances the integrity of the epithelial barrier in TNBS-induced colitis by increasing mucin and tight junction-related protein secretion. A study that investigated the protective effect of SLBZP on hepatic inflammatory injury in rats with NAFLD through the TLR4/NLRP3 signalling pathway reported that SLBZP inhibited the activation of the NLRP3 inflammatory reaction and the release of interleukin 1b in rats with NAFLD induced by a high-fat diet (HFD) by inhibiting the expression of TLR4 induced by LPS (Pan et al., 2021). Tang et al. (2020) explored the potential mechanism by which SLBZP acts against NAFLD using *in vivo* experiments and found that SLBZP increased the adiponectin level in the liver and serum and inhibited the expression of sterol regulatory element binding protein-1c (SREBP-1c), thus regulating systemic lipid metabolism and reducing lipid accumulation in the liver. Additionally, Zhang et al. (2018) found that the effect of SLBZP on NAFLD may be associated with increased abundance of beneficial intestinal flora and decreased levels of LPS in the portal vein. This medicine thus has the clinical effects of invigorating the spleen and the qi. In recent years, clinical studies have also reported that SLBZP reduces blood and liver lipids (Hua Gang, 2009; Wei Jitong, 2011; Yue-qiao, 2012; Xianzhao et al., 2017), thereby affecting the intestinal flora (Liang, 2017).

In clinical practice, we have found that the development of NAFLD is mostly associated with phlegm, qi stagnation and blood stasis and that its effective treatment is mostly based on strengthening the spleen and draining the liver (Guihong et al., 2019). Within TCM, it is believed that by enhancing the spleen’s ability to transport and transform, the liver’s qi can be regulated, thereby improving fatty deposits in the liver. In TCM, the spleen is considered to be involved in exerting certain functions of the gastrointestinal tract, and the intestinal microecology is a good reflection of the spleen’s ability to transport and transform (Mingbing et al., 2018). Furthermore, liver cells are rich in mitochondria, which are the centre of energy metabolism and are closely related to qi in TCM (Luo et al., 2022).

In this study, we aimed to experimentally determine whether the spleen-strengthening formula SLBZP affects liver mitochondrial function through SCFAs produced by intestinal flora metabolism. We also discuss the relationship between intestinal homeostasis and liver energy metabolism based on the interaction between the intestine and liver to explain the scientific concept of “treating the liver and invigorating the spleen”.

## 2. Materials and methods

### 2.1. Materials and diet

SLBZP contains 10 traditional Chinese herbal medicines (produced by Beijing Tongrentang Pharmaceutical Factory) (production batch number: 18101038). Its specific composition is as follows: ginseng, yam (fried), coix seed (fried), Poria, *Fructus Amomi* (salt-baked), white lentil (fried), licorice, *Platycodonis Radix*, atractylodes and lotus seed. It is taken orally at 6 g thrice daily. SLBZP was prepared into an aqueous decoction, stored, sealed at 4°C and administered by heating in a water bath at 37°C during gavage. Metformin is used as a positive drug group (produced by Guizhou Tianan Pharmaceutical Co., Ltd., production batch number: 201903074).

The HFD consisted of 15% lard +5% sucrose +10% egg yolk powder +2% cholesterol +1% sodium cholate +67.9% basal feed. Quality control of SLBPZ was performed using high-performance liquid chromatography–mass spectrometry. The identification of the SLBZP components was outsourced to Shanghai BIOTREE Biological Technology Co., Ltd. (Supplementary Figure S1).

### 2.2. Animals, grouping, moulding, and drug administration

Forty-eight male C57BL/6 mice (aged 6 weeks) of SPF grade weighing 20 ± 2 g were purchased from the Laboratory Animal Department of Kunming Medical University, Kunming [Certificate No: SCXK (Yunnan) K2015-0002]. This study was conducted in strict accordance with the requirements of Yunnan University of Traditional Chinese Medicine’s Guide to the Care and Use of Experimental Animals. The mice were housed in the animal room of Yunnan University of Traditional Chinese Medicine (Ethics Committee approval number:R-082018046). The breeding environment met the SPF animal environmental standard and had good ventilation, with humidity of 55% ± 10%, temperature of 25°C ± 1°C and lighting involving a 12 h light/12 h dark cycle. The animals were provided with feed *ad libitum*.

The mice were randomly divided into six groups: normal group (*n* = 8), model group (*n* = 8), SLBZP low-dose group (*n* = 8), SLBZP middle-dose group (*n* = 8), SLBZP high-dose group (*n* = 8) and Metformin group (*n* = 8). The normal group was fed a standard diet, whereas the other groups were fed an HFD. Drug intervention was started at the end of the 4th week, with continuous administration for 4 weeks, and all samples were collected at the end of the 8th week.

The NAFLD mouse model was induced using the HFD. Hematoxylin–eosin (H&E) and oil red O staining were used to observe the mouse liver cells. The appearance of fat vacuoles in the liver cells, which accounted for one-third of the slice field, was considered to reflect the successful modelling of NAFLD.

We started the drug intervention at the end of week 4 by gavage according to the body weight of the mice, with an average of 0.2 mL for 20 g.The normal and model groups were administered 0.9% NaCl at 100 mL/kg by gavage; the metformin group was administered 0.9% NaCl-dissolved metformin hydrochloride enteric-coated tablet powder (0.468 g/kg).The low dose group of SLBZP corresponds to clinical equivalent dosage;and the low, middle and high SLBZP groups were administered 1, 2 and 4 times the clinically equivalent amount of SLBZP (2.34, 4.68 and 9.36 g/kg, respectively).

### 2.3. Sample preparation

On the day before euthanasia, we took a stool sample for *16S rDNA* sequencing. After the last administration and fasting for 12 h, all mice were anaesthetised by intraperitoneal injection of 3% pentobarbital (0.1 mL/100 g body weight). After excising the whole liver, a separated part was immediately placed into mitochondrial suspension at 4°C and placed on ice for later use. The remaining liver was stored at −80°C for histological and lipid content analyses. The intestinal contents were stored in a freezer at −80°C for the detection of SCFAs. After collecting blood from the orbital vein with a disposable vacuum blood collection tube, some of the whole blood was used for fasting blood glucose (FBG) detection, while the rest was centrifuged at 3000 × *g* and 4°C for 15 min. Subsequently, the supernatant was collected, distributed into 1.5-mL centrifuge tubes, and stored at −20°C for biochemical analysis.

### 2.4. Biochemical analysis

The liver tissue was minced, homogenised and centrifuged at 3000 × *g* for 10 min at 4°C. Subsequently, the cleared supernatant was collected. The levels of total cholesterol (TC), triglycerides (TG), serum alanine aminotransferase (ALT) and aspartate aminotransferase (AST) in the liver tissue were measured using a Roche automatic biochemical analyser (Roche Company, Switzerland; model: Cobas c311) and a kit (Nanjing Jiancheng Bioengineering). The eyeballs of the mice were harvested, venous blood was collected and whole blood samples were taken for FBG detection using a blood glucose metre. The samples were left for about 2 h and centrifuged at 3000 rpm for 15 min. The supernatant was used for the assessment of liver function and blood lipid indices, including ALT, AST, TC, TG, high-density-lipoprotein cholesterol (HDL-C), low-density-lipoprotein cholesterol (LDL-C) and total bilirubin (TBIL). Each step was performed according to the kit’s instructions.

### 2.5. Pathological examination of liver tissue

The liver was fixed in tissue fixative for >48 h and then removed, trimmed into a 5 × 5 × 2 mm cube and stained with H&E and oil red O stains following the staining kit instructions. Finally, gelatin was used to seal the film, and the histopathological changes in the liver samples were observed under a microscope.

### 2.6. Detection of bacterial abundance in mouse faeces

We collected the mouse faeces, transferred them to a sterilised Eppendorf (EP) tube, froze them in liquid nitrogen and stored them at −80°C. The stored intestinal contents were sent on dry ice to Shanghai Aqu Biotechnology Co., Ltd., for testing (Edgar, 2013; Bolger et al., 2014; Kechin et al., 2017). We upload the original data to NCBI:SRP445755.

DNA was extracted using the QIF DNA SMK (No. 51724) kit in accordance with the manufacturer’s instructions. Subsequently, reagents and primers were added to amplify the V3–V4 region of bacterial RNA genes to obtain amplified fragments of >500 bp. The following primers were used: F: 5′-CCTACGGGRSGCAG-3′; R: 5′-GGACTACVVGGGTATCTAATC-3′. Sequencing was performed using the HiSeq PE250 instrument.

For data processing, the sequences were clustered at the 97% similarity level (USEARCH, v. 10.0), with 0.005% of the number of sequences as the threshold filter OTU. Species annotation RDP Classifier: confidence threshold 0.8; phylogenetic tree analysis multiple comparisons.

### 2.7. Determination of SCFA levels in the intestinal contents of mice

Determination of SCFA levels in mouse colonic contents was performed by Shanghai Aqu Biotechnology as follows: a 50 ± 1 mg sample was placed in 2-mL EP tubes, extracted with 0.5 mL of dH_2_O and vortexed for 10 s. The sample was homogenised in a ball mill for 4 min at 35 Hz and then ultrasound-treated for 5 min (incubated in ice water). It was then centrifuged for 15 min at 10,000 rpm and 4°C. A total of 0.3 mL of supernatant was transferred into a fresh 2 mL EP tube, extracted with 0.5 mL of dH_2_O and vortexed for 10 s. Subsequently, it was homogenised in a ball mill for 4 min at 35 Hz and ultrasound-treated for 5 min (incubated in ice water). It was centrifuged again for 15 min at 10,000 rpm and 4°C. Next, 0.5 mL of supernatant was transferred into a fresh 2 mL EP tube. The supernatant was combined with step 4 for a total of 0.8 mL of supernatant; 0.1 mL of 50% H_2_SO_4_ and 0.8 mL of 2-methylvaleric acid (25 mg/L stock in methyl tert-butyl ether) were added as an internal standard, followed by vortexing for 10 s, with oscillations over 5 min. The sample was centrifuged for 15 min at 10,000 rpm and 4°C and incubated at −20°C for 30 min. The supernatant was transferred into a fresh 2 mL glass vial for GC-MS analysis (Dunn et al., 2011).

### 2.8. Detection of mitochondrial correlates in mouse liver

High calcium sensitivity was measured by adding 1 mL of swelling measurement solution into a reference cup and zeroing the spectrophotometer. One microlitre of mitochondrial suspension was added into the reference cup, blown evenly and put into the measurement chamber. The absorption value (A) was measured at 520 nm every 2 min for 10 min, and the dynamic change in mitochondrial swelling was observed within 10 min (Wieckowski et al., 2009).

Membrane potential was determined as follows: a total of 192 μL of membrane potential reaction substrate solution was added into a 96-well plate, and 4 μL of 26 μmoL/L rhodamine 123 reagent was added and mixed well with a pipette. The fluorescent enzyme standard was adjusted to an excitation wavelength of 503 nm and an emission wavelength of 527 nm, and the basal fluorescence value was measured. Next, 4 μL of mitochondrial suspension was added to the membrane potential reaction matrix solution, mixed with a pipette and left for 30 s at room temperature. Fluorescence was recorded every 30 s for 5 min using the luminescence zymograph to observe dynamic changes in membrane potential.

Total mitochondrial protein concentration [the bicinchoninic acid (BCA) method] was determined by taking a BSA protein standard tube and using protein standard solution to prepare a 0.5 mg/mL protein standard. BCA copper sulfate solution was mixed at 50:1 with an appropriate amount of BCA working solution at 37°C for 30 min, compared with the standard curve No. 0, after which the colorimetric value was determined at a wavelength of 562 nm and the absorbance value of each hole was recorded. A standard curve was prepared for the first row of samples and used to calculate the protein concentration.

Wuhan Xavier Biotechnology performed the determination of liver ATP level. Liquid chromatography-mass spectrometry (LC-MS) was used to measure liver adenosine level. The settings were as follows: chromatographic column (Welch Ultimate XB C18 250 × 4.6 mm, 5 μm); DAD detector, detection wavelength 254 nm; flow rate 1 mL/min; column temperature 30°C; and injection volume 10 μL. The mobile phase was prepared by adding 3.5 mL of reagent I to 1,000 mL of ultrapure water before use. The pH was adjusted to 6.15 with reagent II. The sample was filtered with a 0.22 μm filter membrane and degassed by ultrasonication to form mobile phase A. Mobile phase B was pure acetonitrile with gradient elution. The chromatogram acquisition and integration of the final compounds were processed using the Chemstation software (Gika et al., 2012).

The oxygen consumption of mouse liver mitochondria was measured as follows: oxygen consumption was measured using a biological tissue oxygen consumption measuring instrument and oxygen consumption was calculated based on the results. After calibration, the reaction chamber was carefully washed with water until no powder remained. A total of 2 mL of reaction substrate solution was added to the reaction chamber under magnetic stirring at 100 r/min until the oxygen baseline level was stable. Subsequently, 100 μL of mitochondrial suspension was added. Measurements were performed continuously for 1–1.5 min, oxygen consumption and consumption time were recorded and the state IV respiratory rate (ST4) was calculated. Next, 5 μL of 50 mmol/L ADP solution was added into the reaction chamber and measurements were performed continuously for 10 min, with the oxygen consumption and consumption time being recorded. Finally, the state III respiratory rate (ST3) was calculated.

### 2.9. Western blot detection of mouse liver UCP2, AMPK, and IF1 protein expression

After the liver tissues of each group of mice had been lysed using RIPA lysis solution, total protein from the liver tissues was extracted and the protein level was determined using a BCA kit. After separating the proteins *via* SDS-PAGE electrophoresis, they were electrotransferred to a PVDF membrane. After blocking the proteins at room temperature for 2 h using 5% skim milk, the primary antibodies UCP2 (1:500), AMPK (1:500) and IF1 (1:1000) at appropriate concentrations were incubated overnight at 4°C. On the second day, the secondary antibody was incubated with TBST (washing buffer) for 1 h at room temperature. The membrane was washed with TBST to visualise the protein. Alpha software was used to quantify the grayscale values of the protein bands using actin as an internal reference.

### 2.10. Statistical analysis

Statistical analysis was performed using SPSS v.22.0, and the data are expressed as mean ± standard deviation (x̅ ± s). The homogeneity of variance was compared among groups, and univariate analysis was used for comparisons among groups. The rank sum test was used for variance heterogeneity, the LSD test was used for variance homogeneity and Dunnett’s T3 test was used for uneven variance. A *p*-value of <0.05 was considered statistically significant.

## 3. Results

### 3.1. SLBZP reduces the body weight and liver-to-body ratio of HFD-fed mice

**Figure 1:**
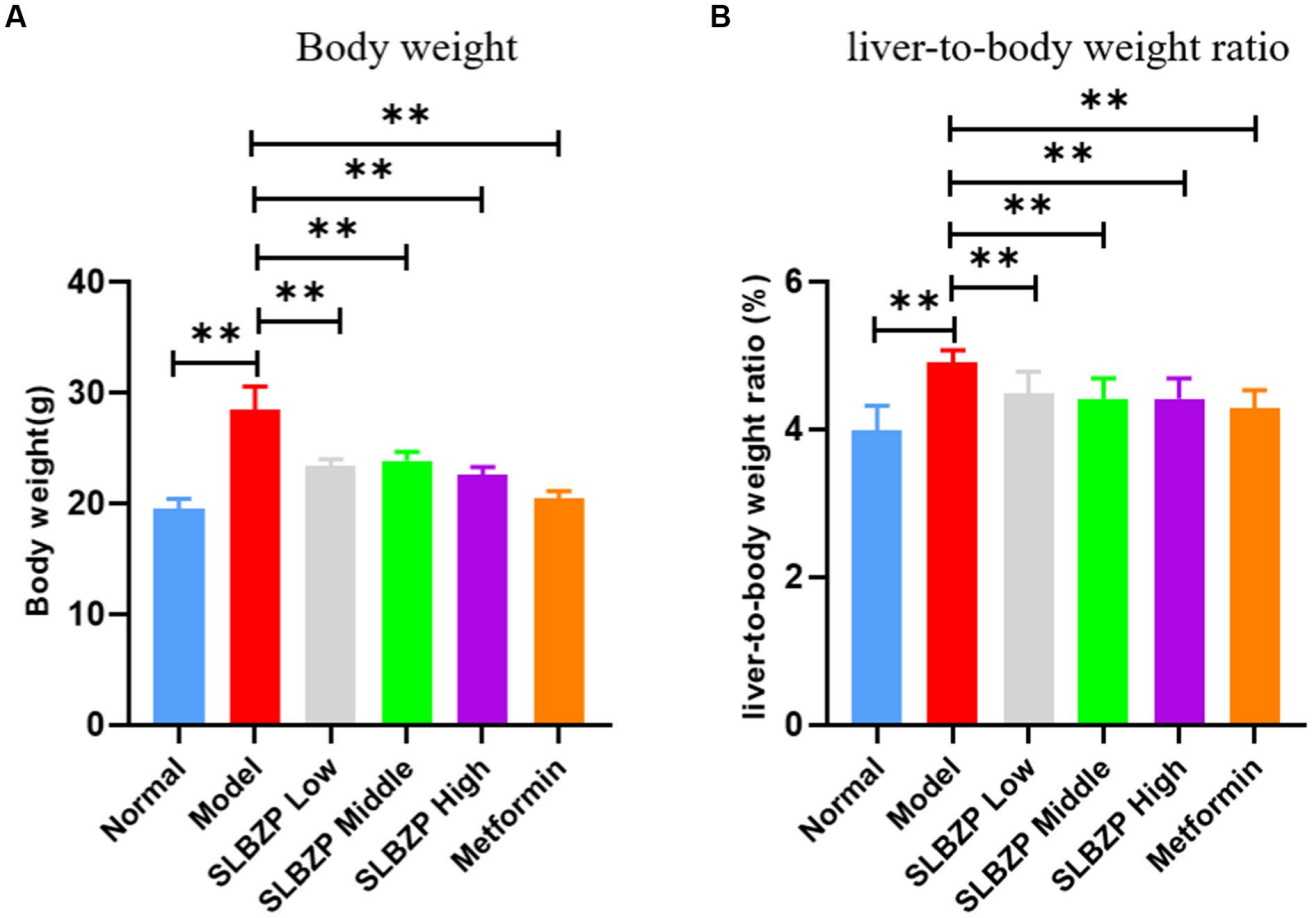
Change in Body weight **(A)** and liver-to-body weight ratio **(B)** of mice in each group during the experiment. Compared with the model group (*n* = 8 per group),^*^p < 0.05, ^**^p < 0.01.

The weights of the mice in the model group were significantly higher than those in the normal group (*p* < 0.01). Compared with the model group, the weights of the mice in each drug group were significantly decreased (*p* < 0.01). The liver-to-body ratio of mice in the model group was significantly higher than that of mice in the normal group (*p* < 0.01), and the liver-to-body ratio of mice in each group was significantly lower than that of mice in the model group (*p* < 0.01; Figures 1A,B).

### 3.2. Effect of SLBZP on pathological changes in the liver of HFD-fed mice

**Figure 2:**
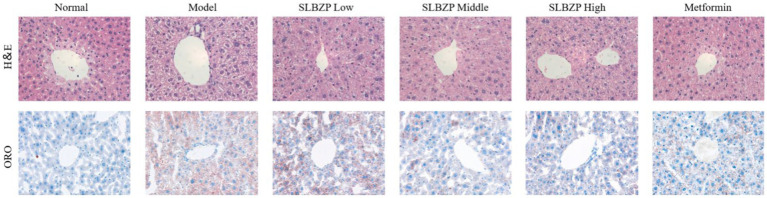
H&E and oil red O staining. All tissues were observed using a light microscope; representative images are shown (×400).

In the 8th week, the liver of the normal group is bright red with a smooth surface. Compared with the normal group, fat vacuoles appeared in the model group, the arrangement of hepatic cords was disordered, some liver cells were ruptured, the colour of the liver nuclei was deepened after staining and inflammatory factors infiltrated the area. Compared with the model group, the structure of hepatocytes, the arrangement of hepatic cords and the structure of hepatic lobules in the SLBZP low-dose group were not significantly abnormal. In the SLBZP middle-dose group, scattered small fat vacuoles and clear hepatic cords were observed. In the SLBZP high-dose group, small fat vacuoles were observed, which were smaller and fewer than those in the model group. In the metformin group, a small number of fat vacuoles were observed, the morphology of liver cells was normal and the arrangement of the hepatic cords was regular. The liver injury of each drug treatment group was less severe than that of the model group, with the middle-dose SLBZP group exhibiting the most apparent effect (Figure 2).

### 3.3. SLBZP improves the lipid profile and reduces liver injury in HFD-fed mice

**Figure 3:**
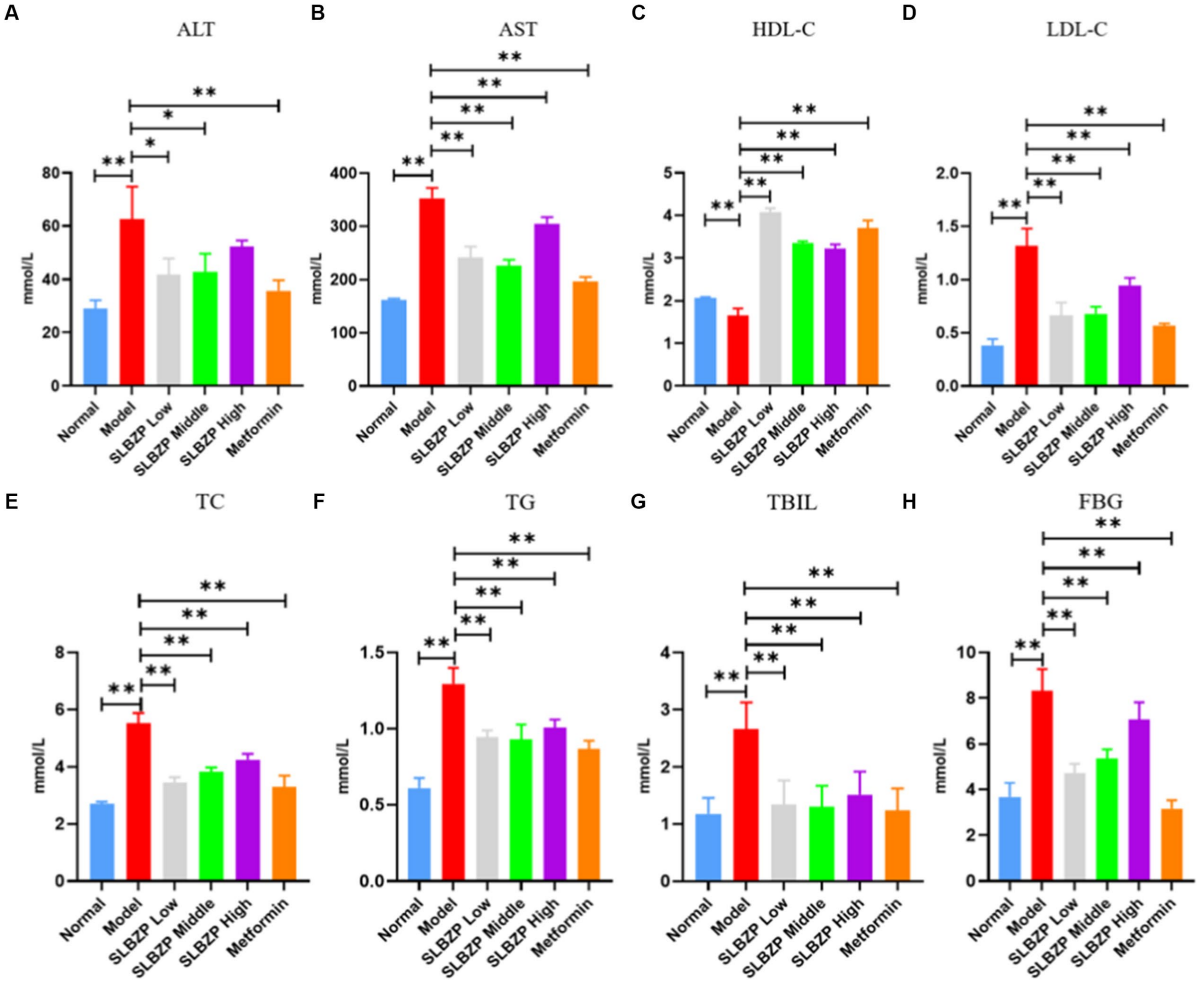
Serum biochemical indices of mice in each group **(A)** ALT, **(B)** AST, **(C)** HDL-C, **(D)** LDL-C, **(E)** TC, **(F)** TG, **(G)** TBIL, **(H)** FBG. Compared with the model group (*n* = 8), ^*^p < 0.05, ^**^p < 0.01..

Regarding liver function and TBIL, compared with the levels in the normal group, serum ALT, AST and TBIL levels in the model group were significantly increased (*p* < 0.01). Moreover, compared with the levels in the model group, ALT, AST and TBIL levels in the metformin group were significantly decreased (*p* < 0.01). Furthermore, AST and TBIL levels were significantly decreased (*p* < 0.01) and ALT, AST and TBIL levels were significantly decreased (*p* < 0.05) in the SLBZP low-dose and middle-dose groups (*p* < 0.01), respectively. ALT and TBIL levels in the SLBZP high-dose group were significantly decreased (*p* < 0.01). Regarding fasting blood glucose, compared with the level in the normal group, the serum FBG level in the model group was significantly increased (*p* < 0.01). Additionally, the serum FBG level in all treatment groups was significantly decreased (*p* < 0.01). Regarding blood lipid levels, compared with the levels in the normal group, serum TC, TG and LDL-C levels in the model group were significantly increased, whereas HDL-C level was significantly decreased (*p* < 0.01). Compared with the levels in the model group, serum TC, TG and LDL-C levels in all drug groups were significantly decreased, whereas HDL-C level was significantly increased (*p* < 0.01) (Figure 3).

### 3.4. Effect of SLBZP on intestinal flora at the phylum level and relative abundance at the genus level

**Figure 4:**
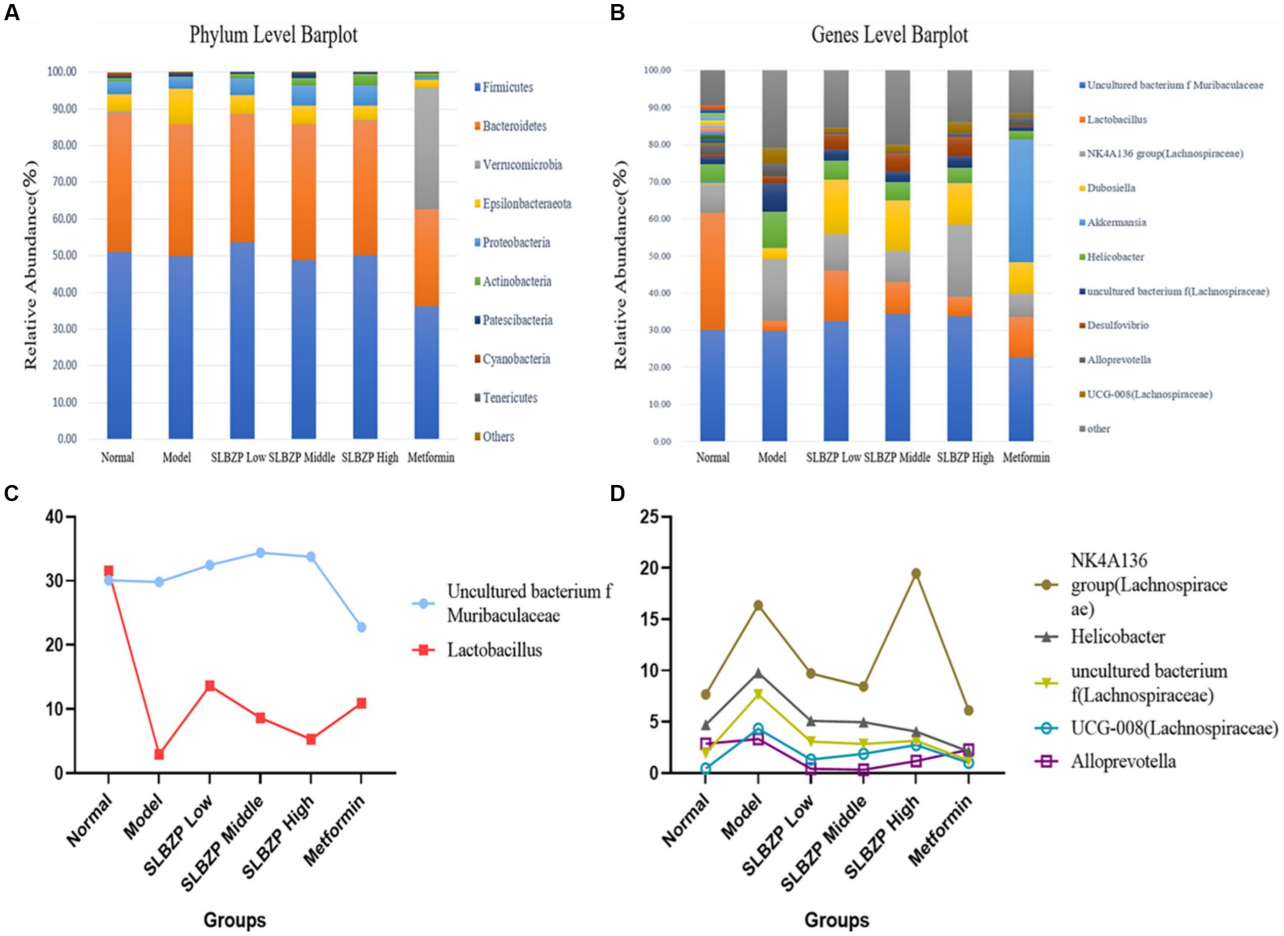
Detection of intestinal flora in mice. **(A)** Relative abundance of the 10 species with the highest relative abundance at the phylum level. **(B)** Relative abundance of the 10 species with the highest relative abundance at the genus level. **(C)** Two bacterial genera whose relative abundance increased after SLBZP intervention. **(D)** Five bacterial genera with reduced relative abundance after SLBZP intervention (*n* = 8).

Compared with the findings in the normal group, the relative abundances of two phyla in the model group increased (mainly Epsilonbacteraeota) while those of eight phyla decreased (mainly Firmicutes). Compared with the findings in the model group, the relative abundances of two phyla in the SLBZP intervention groups increased (mainly Proteobacteria) and the relative abundances of two phyla in the metformin group increased (mainly Verrucomicrobia) while those of eight phyla decreased (mainly Firmicutes). Meanwhile, in the low-dose group, the relative abundances of four phyla increased (mainly Bacteroidetes) while those of six phyla decreased (mainly Firmicutes) compared with the middle-dose group. In the middle-dose group, the relative abundances of three phyla increased (mainly Firmicutes) while those of six phyla decreased (mainly Bacteroidetes) compared with the high-dose group. Five phyla in the high-dose group exhibited increased relative abundances (mainly Bacteroidetes) and four exhibited decreased relative abundances (mainly Firmicutes). The distribution of Bacteroidetes and Firmicutes in the low, middle and high-dose groups was more similar to that in the normal group (Figure 4A).

Compared with the relative abundance in the normal group, the intestinal flora of mice in the model group exhibited an increase in the abundance of seven genera [dominated by the Lachnospiraceae *NK4A136* group, *Dubosiella*, *Helicobacter* and uncultured bacterium f (*Lachnospiraceae*)] and a decrease in the abundance of three genera (dominated by *Lactobacillus* and uncultured_bacterium_f_*Muribaculaceae*). Compared with the findings in the model group, the relative abundances of four genera of uncultured_bacterium_f*_Muribaculaceae*, *Lactobacillus* and *Duchenne* increased while those of four genera of *Helicobacter* decreased in the SLBZP intervention groups. The relative abundances of three genera of *Lactobacillus* increased while those of three genera of *Helicobacter* decreased in the metformin group. In the metformin group, the relative abundances of three genera of *Lactobacillus* increased while those of seven genera of *Helicobacter* and *Lachnospiraceae_NK4A136_group* decreased. Compared with the SLBZP middle-dose group, the relative abundances of three genera increased (dominated by uncultured_bacterium_f*_Muribaculaceae* and *Desulfovibrio*) while those of seven genera (dominated by Lachnospiraceae *NK4A136* group, *Dubosiella* and *Lactobacillus*) decreased in the SLBZP low-dose group. The relative abundances of six genera (dominated by Lachnospiraceae *NK4A136* group) and four genera (dominated by *Lactobacillus* and uncultured_bacterium*_f_Muribaculaceae*) increased in the SLBZP high-dose group compared with that in the SLBZP medium-dose group. Compared with the SLBZP low-dose group, in the SLBZP high-dose group, the relative abundances of seven genera (mainly uncultured_bacterium_f_*Muribaculaceae* and *Lachnospiraceae NK4A136* group) increased while those of three genera (*Lactobacillus*, *Dubosiella* and *Helicobacter*) decreased (Figure 4B).

The relative abundance of uncultured_bacterium_f*_Muribaculaceae* and *Lactobacillus* decreased after modelling and increased after drug intervention (Figure 4C); conversely, the relative abundance of the *NK4A136* group, *Helicobacter* uncultured bacterium f *(Lachnospiraceae)*, *UCG-008 (Lachnospiraceae)* and *Alloprevotella* increased after modelling and decreased after drug intervention (Figure 4D).

### 3.5. Effect of SLBZP on intestinal flora diversity in HFD-fed mice

**Figure 5:**
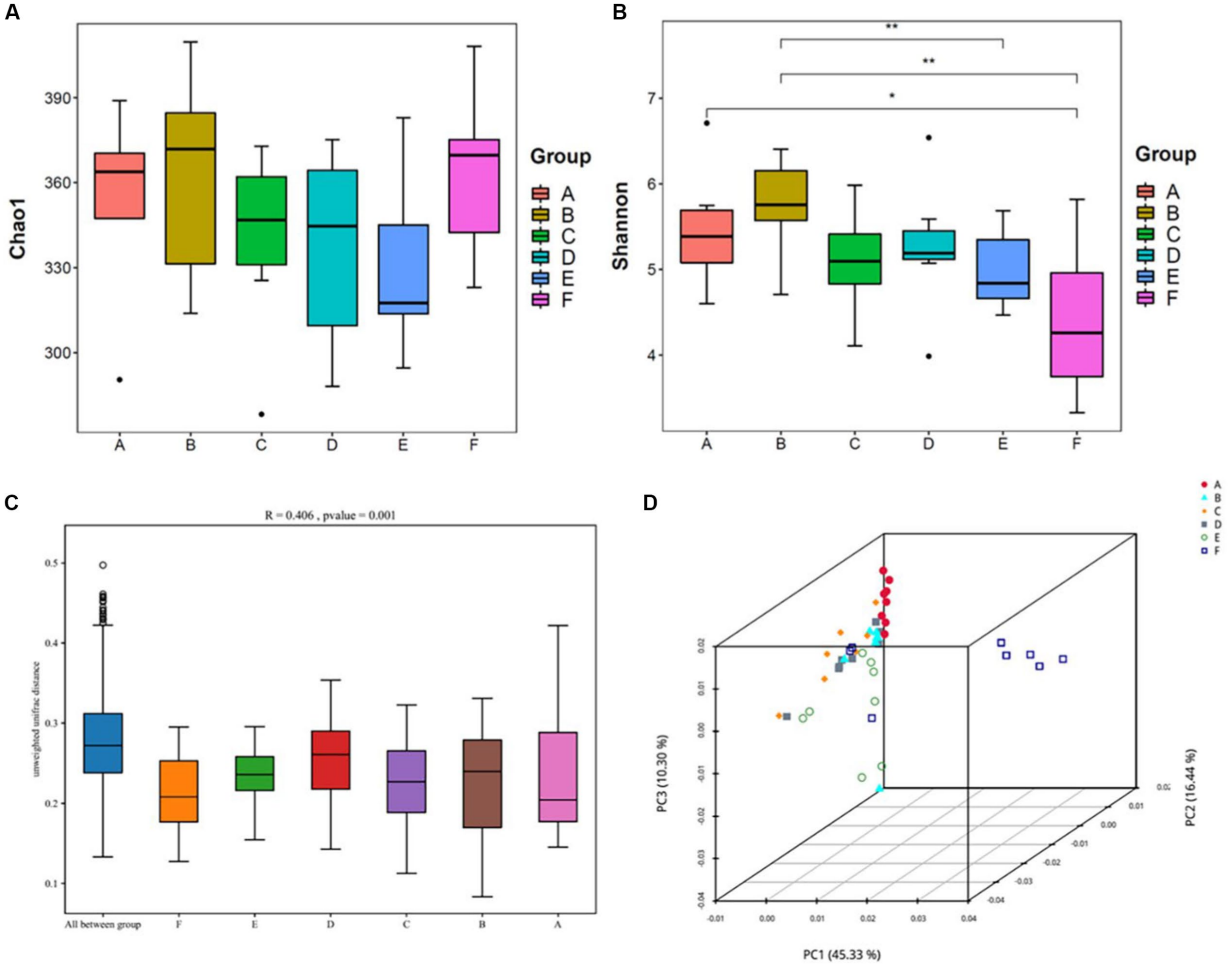
Analysis of the diversity of the intestinal contents of mice(*n* = 8) **(A,B)** Shows the Chao1 and Shannon indices from the α-diversity analysis; **(C,D)** shows the β-diversity analysis, with **(C)** being the Anosim analysis: the vertical coordinates represent β-distances; the box plot above “All between^*^” represents β-distance data for all samples between groups, and the box plots after that show β-distance data between samples within groups for different groupings. **(D)** Shows the principal coordinate analysis. A: normal, B: model, C: SLBZP low, D: SLBZP middle, E: SLBZP high, F: metformin.

Alpha diversity reflects the abundance and diversity of a single sample and is measured by various indices, including the Chao1 index for species abundance and the Shannon index for species diversity. Herein, QIIME software was used to calculate the α-diversity index of the samples; estimate the total number of operational taxonomic units contained in the samples based on the Chao1 index values; and estimate the diversity of the microbial communities based on the Shannon index values (Figure 5).

#### 3.5.1. Chao1 index

Compared with the normal group, the Chao1 indices of the intestinal contents of mice in the model and metformin groups increased, while those in the SLBZP dose groups slightly decreased. (Figures 5A,B)

#### 3.5.2. Shannon index

Compared with the normal group, the Shannon index of the intestinal contents of mice in the model group increased, while the indices in the SLBZP and metformin groups decreased, and the difference between the metformin and normal groups was statistically significant (*p* < 0.05).

β-diversity analysis of the intestinal flora was performed using the Unifrac distance method to calculate the distance between samples and to compare the magnitude of differences in species diversity between samples and groups. Anosim analysis yielded an *R*-value of 0.406 and a *p*-value of 0.001, suggesting that between-group differences in the intestinal flora contents of the mice were greater than within-group differences and were statistically significant (Figure 5C). In principal coordinate analysis (Figure 5D), the normal and other groups of intestinal flora of mice were found to be gradually separated, and the SLBZP intervention groups did not overlap with the normal group; however, there was a tendency for them to approach it. Moreover, there is still overlap between the drug treatment group and the model group. Therefore, we speculated that after 8 weeks of HFD feeding and drug intervention, the intestinal flora structure of mice in each group showed some differences, and the SLBZP improved the HFD-induced alteration of the flora structure.

### 3.6. Effect of SLBZP on SCFA levels in the intestinal contents of HFD-fed mice

**Figure 6:**
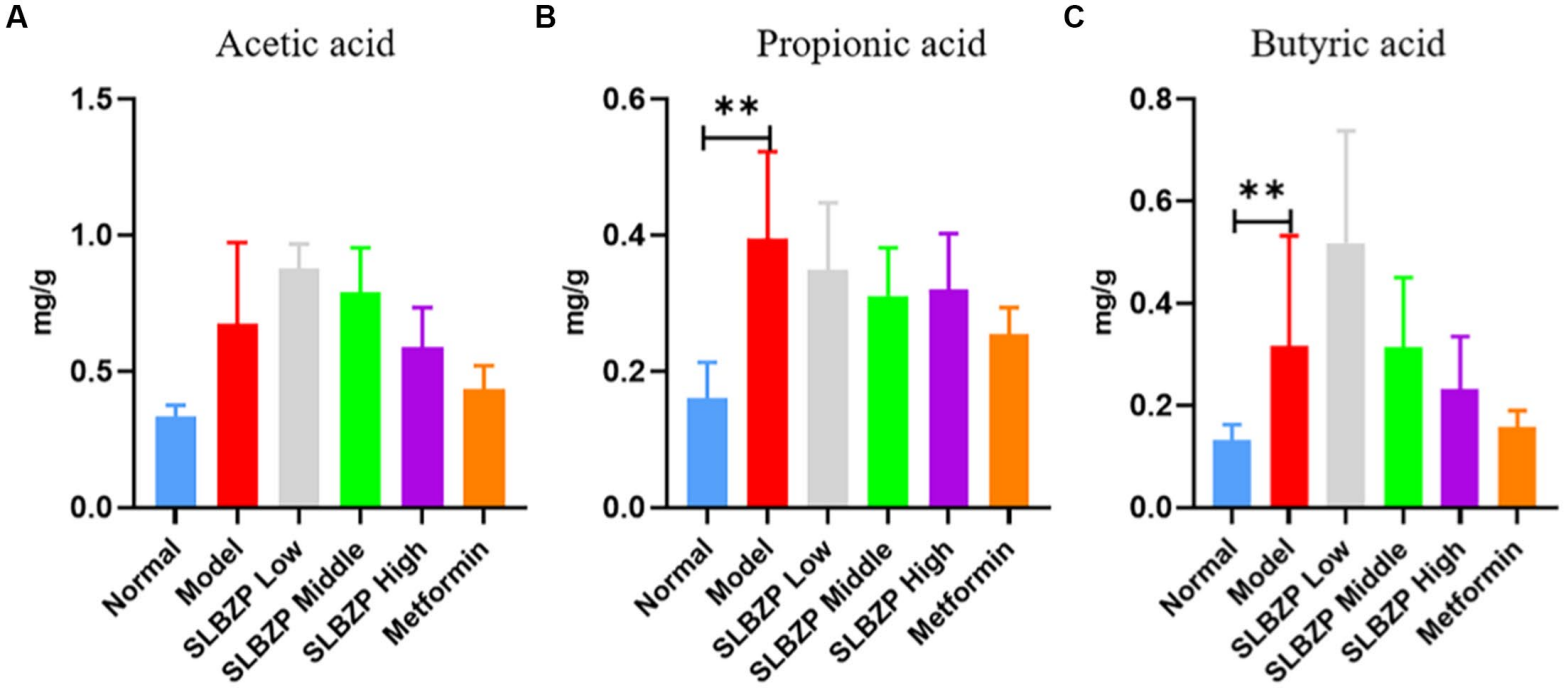
Changes in short-chain fatty acid levels in mice **(A)** Acetic acid, **(B)** Propionic acid, **(C)** Butyric acid (*n* = 6); ^*^p < 0.05, ^**^p < 0.01.

Compared with the normal group, the levels of propionic and butyric acids in the model group significantly increased (*p* < 0.01). Compared with the model group, the level of propionic acid in the SLBZP low-, middle-and high-dose groups and the metformin group showed a downward trend (*p* > 0.05), but there was no significant change in the levels of acetic and butyric acids (Figure 6).

### 3.7. Correlation between intestinal flora and environmental factors at the genus level in SLBZP-treated HFD-fed mice

**Figure 7:**
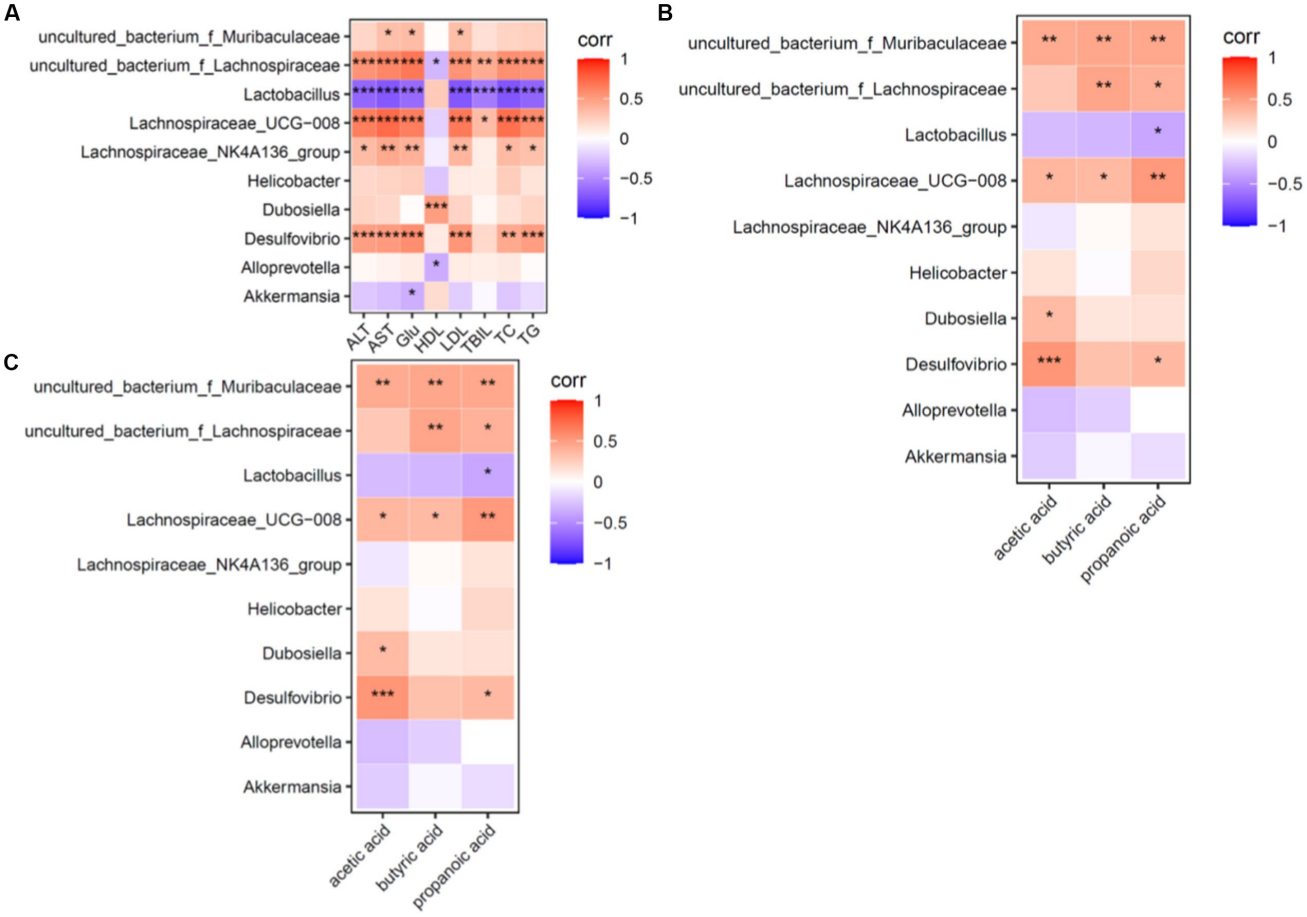
Heat map for correlation analysis of serum biochemical indices, dominant genera and short-chain fatty acids in mice (*n* = 6). **(A)** Shows a heat map of correlation analysis between serum biochemical indices and dominant intestinal bacterial genera in mice. **(B)** Shows the heat map of correlation analysis between intestinal short-chain fatty acids and dominant intestinal bacterial genera in mice. **(C)** Shows the heat map of correlation analysis between dominant bacterial genera and short-chain fatty acids in mice; red (corr = 1), blue (corr = −1) and white (corr = 0); data with a correlation *p*-value of <0.05 are marked with “^*^” in the graph. The vertical coordinates are the differential flora and the horizontal coordinates are the differential metabolites. Five biochemical indicators and three metabolites were closely associated with the dominant genus.

The correlations between the dominant genus and ALT, AST, TC, TG, LDL-C, HDL-C, TBIL, and SCFA levels were made at the genus level, and a heat map was plotted to show the correlations between the dominant genus and the above environmental factors (Figure 7).

#### 3.7.1. Correlation factors

Correlation factors: ALT, AST, TC, TG and LDL-C levels were positively correlated (*p* < 0.05) with uncultured_bacterium_f_*Lachnospiraceae*, *Lachnospiraceae*_UCG-008 and *Desulfovibrio* and were negatively correlated (*p* < 0.05) with *Lactobacillus* (*Lactobacillus* spp). Based on these findings, ALT, AST, TC, TG and LDL-C levels were the factors closely related to intestinal flora structure.

#### 3.7.2. Dominant genera

Dominant genera: Uncultured bacterium_f_*Muribaculaceae*, uncultured bacterium_f_*Lachnospiraceae*, *Lachnospiraceae* UCG-008, *Desulfovibrio*, *Lachnospiraceae*_f_*Lachnospiraceae* and *Lachnospiraceae*_NK4A136_group were positively correlated (*p* < 0.05) and *Lactobacillus* (*Lactobacillus* spp.) was negatively correlated (*p* < 0.05) with the environmental factors of this experiment. It can be seen that uncultured bacterium_f_*Muribaculaceae* genus, uncultured bacterium_f_*Lachnospiraceae* genus, *Lachnospiraceae* UCG-008 genus, *Desulfovibrio* genus, *Lachnospiraceae*_NK4A136_group and *Lactobacillus* (genus *Lactobacillus*) are the dominant genera of interest in this experiment.

### 3.8. Effect of SLBZP on indices related to liver mitochondria in HFD-fed mice

**Figure 8:**
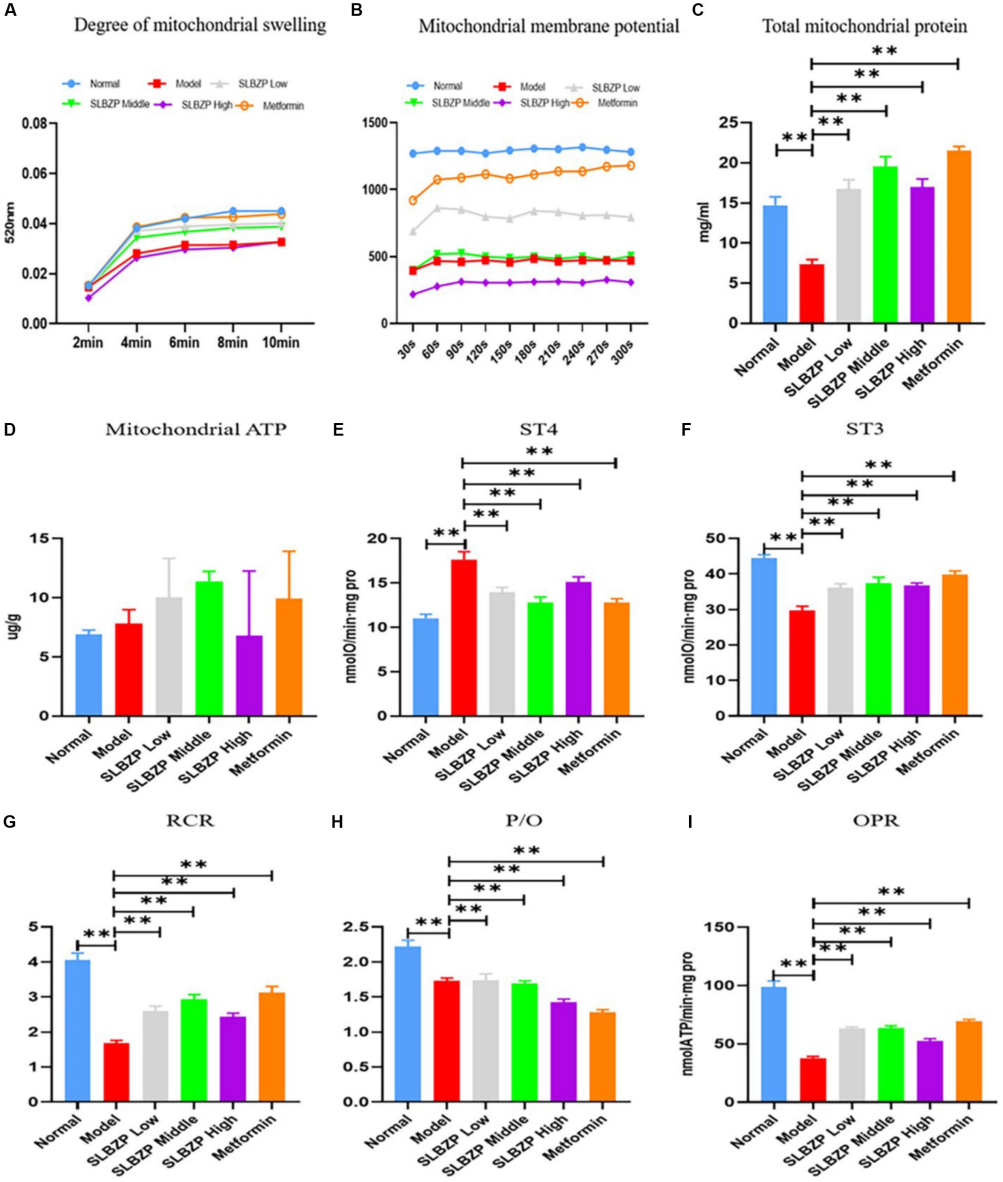
Related indices of liver mitochondria in mice. **(A)** Swelling of liver mitochondria in a high calcium environment at different time points (*n* = 8). **(B)** At different time points, the mitochondrial membrane potential of the liver in each group was measured (*n* = 8). **(C)** The total protein concentration of liver mitochondria in each group at different time points (*n* = 8). **(D)** ATP level in the liver of each group (*n* = 6). **(E–I)** The oxygen consumption of liver mitochondria in each group. Compared with the model group (*n* = 8), ^*^p < 0.05, ^**^p < 0.01.

Compared with the normal group, the absorbance values of mitochondria at A520 nm were reduced in all of the other groups, indicating impaired mitochondrial membrane function. The absorbance value of the model group was the smallest among the groups, and the Metformin group was closest to the absorbance value of the normal group, followed by the SLBZP low-dose, middle-dose and high-dose groups; however, the differences were not statistically significant (Figure 8A). The liver mitochondrial membrane potential assay results of the metformin group were the closest to those of the normal group in terms of fluorescence value, followed by the SLBZP low-dose, middle-dose and high-dose groups (Figure 8B).

Compared with the normal group, the liver mitochondrial protein level in the model group was significantly decreased. Compared with the model group, the liver mitochondrial protein level was significantly increased in the other drug groups (*p* < 0.01; Figure 8C). Furthermore, compared with the model group, ATP levels were significantly increased in the metformin and SLBZP low-dose and middle-dose groups (Figure 8D).

The ST4 value was significantly higher in the model group than in the normal group. The ST3 value, mitochondrial respiratory control ratio (RCR), phosphorus oxygen ratio (P/O) and oxidative phosphorylation rate (OPR) were significantly lower (*p* < 0.01). Compared with the model group, the ST4 value was significantly lower and ST3, RCR, P/O and OPR were significantly higher in each drug group (*p* < 0.01; Figures 8E–I).

### 3.9. Effect of SLBZP on the hepatic UCP2/AMPK/IF1 pathway in HFD-fed mice

**Figure 9:**
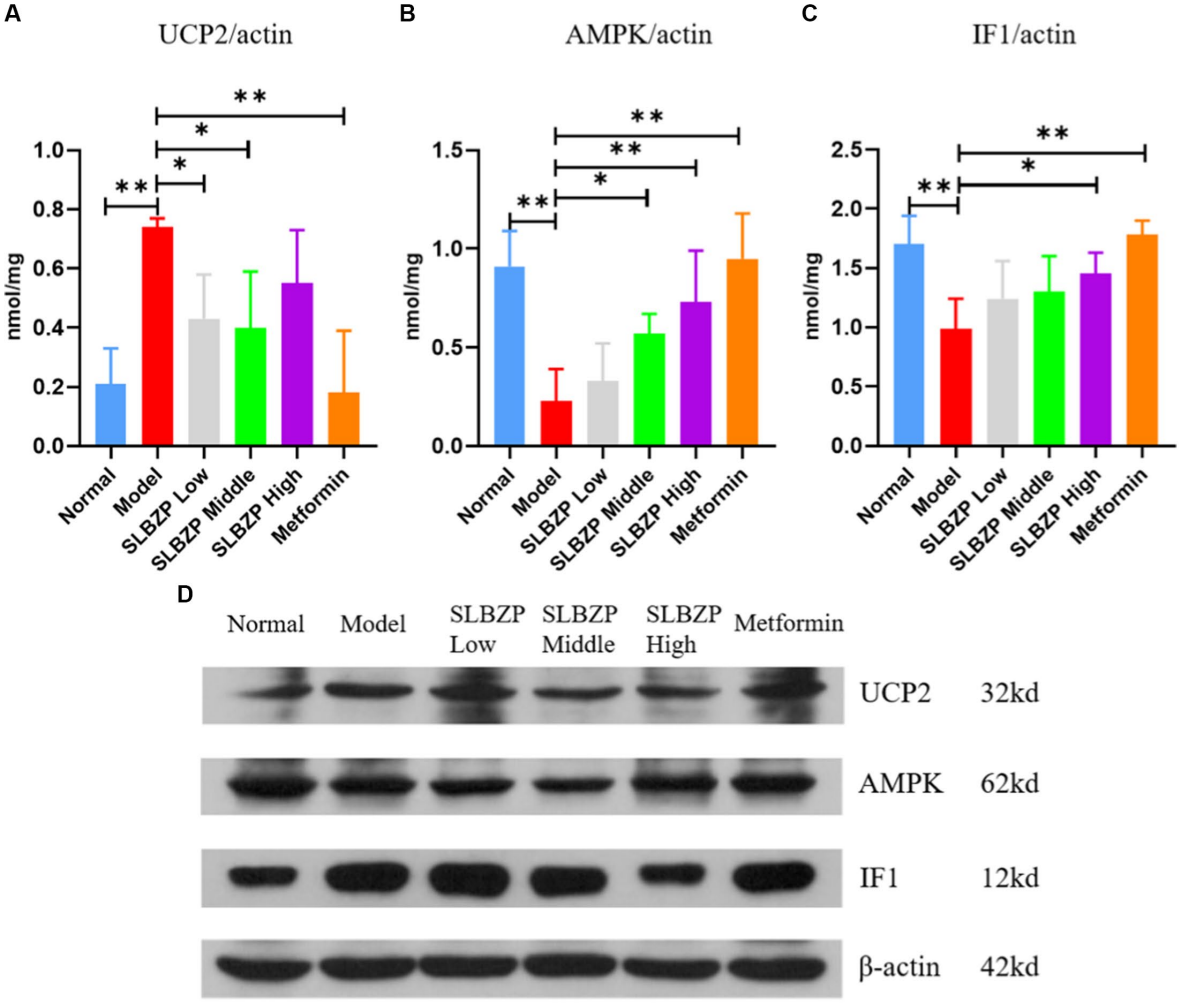
Results of UCP2, AMPK and IF1 protein expression in mouse liver. **(A–C)** Protein expression level of UCP2, AMPK and IF1 in each group. **(D)** Western blotting was used to analyse the expression of UCP2, AMPK and IF1 in the liver tissue of mice in each group. Compared with the model group (*n* = 3), ^*^p < 0.05, ^**^p < 0.01.

As shown in Figure 9, compared with the normal group, UCP2 expression was significantly increased in the model group (*p* < 0.01). Compared with the model group, UCP2 expression in the SLBZP low-dose and middle-dose groups was decreased (*p* < 0.05). In the metformin group, UCP2 expression was significantly decreased (*p* < 0.01). Figure 1B shows that compared with the normal group, AMPK expression was significantly decreased in the model group (*p* < 0.01). Compared with the model group, AMPK expression was significantly increased in the metformin and SLBZP high-dose groups (*p* < 0.01) and AMPK expression was elevated in the SLBZP middle-dose group (*p* < 0.05). Figure 1C shows that compared with the normal group, IF1 expression was significantly increased in the model group (*p* < 0.01). Additionally, compared with the model group, IF1 expression was significantly increased in the metformin (*p* < 0.01) and SLBZP high-dose (*p* < 0.05) groups.

## 4. Discussion

SLBZP consists of ginseng, atractylodes, Poria, yam, lotus seeds, white lentils, coix seeds and other ingredients. It was shown in a previous study that atractylodes, *Poria cocos* and licorice have important effects on the regulation of intestinal flora and SCFAs in spleen-deficient rats (Wen-wu et al., 2019). Yam was also shown to alleviate antibiotic-associated diarrhoea, alter the intestinal microbiota and increase the levels of SCFAs in mice (Zhang N. et al., 2019).The active ingredients in SLBZP impact the intestinal flora and are anti-inflammatory. Studies have found that atractylodes polysaccharides and atractylenolide improve intestinal flora dysbiosis in rats (Wang et al., 2014; Feng et al., 2020). Atractylenolides I, II and III have also been reported to have anti-inflammatory effects (Wei et al., 2006a,b). Several recent studies have shown that ginsenosides can be metabolised by the intestinal flora into deglycosylated ginsenosides, which are then absorbed into the blood (Dong et al., 2017). The intestinal flora also promotes the absorption and metabolic conversion of the ginsenosides Rb1 and Rd (Kim et al., 2014). One study (Qi et al., 2022) found that total ginsenosides ameliorated lipopolysaccharide-induced acute lung injury in mice by modulating intestinal flora and SCFA metabolism. Another study found that gavage with high doses of Poria aqueous extract to mice resulted in a significant increase in the number of bifidobacteria in the intestinal tract, suggesting that Poria has a regulatory effect on the intestinal flora (Song et al., 2011). Poria polysaccharides also have an inhibitory effect on acute and chronic inflammatory responses (Anji et al., 2003). Yam polysaccharides have various biological effects, such as antioxidative (Zhou et al., 2021), hypolipidemic (Fei et al., 2017), antitumor (Shi et al., 2016) and antiageing effects (Wang et al., 2020) as well immunomodulatory activity (Ma et al., 2017). Yam polysaccharides also have significant effects on growth performance and intestinal flora composition in mice and can improve insulin resistance (Qiyu et al., 2015). Additionally, they exert hypolipidemic effects by reducing LDL-C and TC levels, inhibiting the production of very long-chain fatty acids, reducing inflammatory protein products and inhibiting the production of TNF-α (Cheng et al., 2019). Licorice possesses quercetin, a naturally occurring flavonoid with various bioactive effects, such as antioxidant, anti-inflammatory (Loke et al., 2008) and antibacterial effects (Wei et al., 2006a,b). Quercetin also regulates lipid homeostasis, improves intestinal flora composition, regulates hepatic lipid metabolism protein expression, promotes hepatic lipid metabolism and reduces lipid accumulation (Porras et al., 2017; Peng et al., 2020). Quercetin regulates the imbalanced state of intestinal flora, increasing the abundance of *Bacteroides* and decreasing that of *Proteus*, indicating its ability to restore the host–microbial balance (Porras-Sanabria et al., 2016).

This study confirmed that SLBZP improves the pathological state of mice with HFD-induced NAFLD. Compared with the normal group, the HFD group exhibited symptoms of NAFLD, including obesity, liver function damage, liver lipid deposition and hepatitis. SLBZP significantly reduced body weight; FBG and serum AST, ALT and TC levels; and TG and TC accumulation in the liver. In terms of liver pathology, SLBZP improves liver lipid deposition. ALT mainly reflects damage to the hepatocyte membrane and AST mainly reflects damage to mitochondria in hepatocytes. TBIL is the sum of indirect and direct bilirubin and is an important indicator of liver function (Zhu et al., 2015). Our results showed that ALT, AST and TBIL levels in the model group were higher than those in the normal group, indicating that liver cells and the liver mitochondria of NAFLD mice were damaged.

The liver is exposed to intestinal flora metabolites transported through the portal vein. An imbalance of intestinal flora is associated with liver diseases such as NAFLD (Iannucci et al., 2016). Recent studies have shown that SCFAs, as metabolites of intestinal flora, may play a role in NAFLD by affecting inflammation, immunity and mitochondrial metabolism (Mortensen et al., 1994; Haque and Barritt, 2016; Jian’gao, 2016). However, their mechanism of action remains unclear. Some researchers have asserted that the metabolites of intestinal flora enhance mitochondrial activity, improving energy metabolism, such the interaction between the intestinal flora and mitochondria affects the host’s health (Xiuwen, 2017).

According to the modern medicine concept of the gut–liver axis, when the intestinal barrier function is damaged, transfer of intestinal bacteria outside the intestinal cavity, endotoxin enters the portal vein system, the immune system in the liver is activated and inflammatory factors are released, thus damaging the intestinal mucosa and other organs. Therefore, through the gut–liver axis, the gut and liver are closely related physiologically, anatomically and functionally, and they influence each other, affecting the process of liver disease (Miele et al., 2009; Vanni and Bugianesi, 2009). The gut is likely the main target of metformin, not the liver (Bonora et al., 1984; Bailey et al., 1994; Stepensky et al., 2002). In HFD-fed mice, the abundance of *Bacteroides*, one of the *Bacteroides*, was observed to increase upon metformin treatment (Lee and Ko, 2014; Lee et al., 2018; Ryan et al., 2020). With an increase in the abundance of *Bacteroides*, the level of SCFAs in the faeces of metformin-treated mice was higher than that in the faeces of db/db mice (Zhang W. et al., 2019). Some studies have also shown that the levels of tight junction proteins, such as zonulin-1 and occludin, are restored after metformin treatment (Brandt et al., 2019; Ahmadi et al., 2020), while intestinal permeability decreases (Liu et al., 2020). Our experimental results showed that compared with the normal group, the relative abundance of seven genera in the intestinal contents of mice in the model group increased and that of three genera decreased. After drug intervention, the relative abundances of four genera in the SLBZP intervention groups increased while those of four genera decreased. The relative abundance of *Lactobacillus* and *Bifidobacterium* decreased after modelling but increased after drug intervention. Meanwhile, the relative abundance of *Spirillum* increased after modelling but decreased after drug intervention. This shows that SLBZP acts on some genera, which may affect the occurrence and development of NAFLD.

SCFAs involved in human metabolism mainly come from the intestinal flora (Deehan et al., 2020). Over 90% of SCFAs are rapidly absorbed by the intestine and can enter the liver *via* the portal vein (den Besten et al., 2013b). SCFAs play a key role in adipogenesis (McNeil, 1984). Acetic, propionic and butyric acids affect the metabolism of cholesterol, free fatty acids and glucose (den Besten et al., 2013a) to reduce the accumulation of fat in the human body. Specifically, propionic acid increases fat production and glucose intake in the human body (Al-Lahham et al., 2012). Propionic acid also increases the expression of leptin and decreases that of the proinflammatory factor resistin (Al-Lahham et al., 2010). Activation of propionic acid stimulates leptin production in mouse adipocytes (Xiong et al., 2004) to reduce cholesterol synthesis in the liver and improve lipid metabolism (Berggren et al., 1996; Henningsson et al., 2001). Butyric acid regulates adaptive immune cells, protects the intestinal mucosal barrier, prevents harmful substances from invading the human body and inhibits the synthesis of proinflammatory cytokines and mediators (Vinolo et al., 2011). Various bacteria in the intestinal lumen can produce SCFAs by participating in metabolic processes in the intestine. In the intestinal flora, the phylum Firmicutes (gram-positive) was found to be the main SCFA-producing bacterial group, accounting for about 60% of production, while the phylum Bacteroides (gram-negative) accounted for 20%. The phylum Firmicutes (*Clostridium*, *Enterococcus thiercelin* and *Jouhaud*, *Lactococcus lactis*, *Staphylococcus*, *Acetobacter*, *Lactobacillus*, *Bacillus*, etc.) is mainly butyrate-producing. Meanwhile, the phylum Bacteroides (*Bacteroides caccae*, *Bacteroides merdae*, *Bacteroides wdgatus*, *Bacteroides distasonis*, *Bacteroides capillosus*, *Bacteroides IIIfiformis*, etc.) is mainly acetate-and propionate-producing (El Kaoutari et al., 2013; Flint et al., 2015; Levy et al., 2016). SCFAs maintain an acidic environment in the intestinal lumen. This promotes the proliferation of probiotics such as *Bifidobacterium*, *Lactobacillus* and *Bacteroides* and inhibits the growth of pathogenic bacteria such as *Candida*, *Staphylococcus*, *Klebsiella*, *Campylobacter*, *Escherichia coli* and *Bacillus dysenteriae* (Haak et al., 2018). Our results showed that compared with the model group, the intestinal propionic acid levels in the low-, middle-and high-dose SLBZP groups decreased (*p* > 0.05), and acetic and butyric acid levels changed in a dose-response manner. This may be related to the enhanced fermentative capacity of the intestinal microbiota of mice induced by an HFD, with an elevated capacity to produce SCFAs (Murphy et al., 2010). This is consistent with the increased total concentration of SCFAs in the stool of obese individuals reported by Schwiertz et al. (2010). Among them, the levels of acetic and butyric acids continued to increase after drug intervention, which may be related to the relatively high abundance of intestinal lactic acid bacteria in mice (Zhao et al., 2018). After drug intervention, the propionic acid level decreased with a dose–response relationship, but it did not show a significant downward trend, which may be related to the inhibition of propionic acid production caused by an increase in probiotics (He, 2020). These results indicate that SCFAs may be a factor affecting NAFLD development. We also found that compared with the normal group, the propionic and butyric acid levels in the model group increased significantly (*p* < 0.01). Compared with the model group, the propionic acid levels in the low-, middle-and high-dose and metformin groups decreased (*p* > 0.05), but the changes in acetic and butyric acid levels showed no clear trends. Furthermore, analysis of the α-diversity Chao1 and Shannon indices revealed that the abundance and diversity of the flora in the model and normal groups differed from each other, and that the drug-administered groups were closer to the normal group. β-diversity analysis also revealed that the intestinal flora structure of the mice in each group was different, and the SLBZP improved the HFD-induced changes in the flora structure. Correlation analysis of the dominant bacteria at the genus level with biochemical indicators and SCFA levels revealed that ALT, AST, TC, TG, LDL-C and HDL-C levels were correlated with uncultured bacterium_f_*Muribaculaceae* genus, uncultured bacterium_f_*Lachnospiraceae*, *Lachnospiraceae* UCG-008, *Desulfovibrio*, *Lachnospiraceae*_NK4A136_group and other dominant bacteria and SCFAs change into positive correlation. There is a negative correlation with the changes in *Lactobacillus*.

SCFAs also induce UCP2 expression through peroxisome proliferator-activated receptor gamma, which reduces intracellular ATP levels and activates PRKAA1/AMPK signalling pathway molecules, triggering hepatocyte autophagy (Iannucci et al., 2016). UCP2 is located in the mitochondrial membrane of hepatocytes and mediates proton leakage, causing uncoupling of mitochondrial oxidative phosphorylation and energy release as heat, resulting in a decrease in the efficiency of mitochondrial ATP synthesis. AMPK is involved in regulating the metabolism of multiple substances and maintains the balance of cellular energy supply and demand by affecting the metabolism of cellular substances. Furthermore, AMPK is a key regulator of systemic energy homeostasis, which is central to the study of energy metabolic diseases such as obesity, fatty liver and diabetes (Xiaodong, 2002). AMPK affects mitochondrial energy metabolism and promotes ATP synthesis by inhibiting the expression of the mitochondrial ATP synthase inhibitor protein (β-F1 inhibitor protein, IF1; Samari et al., 2005). IF1 inhibits the activity of ATP synthase, the major protein of the mitochondrial oxidative respiratory chain complex V (Wang Yan and Zhijun, 2013).

Mitochondria are organelles with a double-membrane structure, which are found in eukaryotic cells and are the principal site of energy metabolism. The respiratory chain and oxidative phosphorylation are carried out in the mitochondria, and about 90% of ATP in the body is produced in the mitochondria, which are referred to as the “energy factory” of cells. In NAFLD, mitochondria of liver cells exhibit decreased function, along with reduced ATP synthesis and consumption of reactive oxygen species (ROS), which are harmful to cells, but exhibit increased ROS production. Excessive ROS can cause mitochondrial respiratory chain damage, energy metabolism disorder, oxidative stress, lipid peroxidation and destruction of the mitochondrial membrane structure (Andreyev et al., 2020). With the participation of inflammatory mediators such as interleukin-6 and nitric oxide, fatty degeneration, hepatocyte inflammation and necrosis are induced, leading to mitochondria-mediated apoptosis (Skuratovskaia et al., 2021). Mitochondria in NAFLD hepatocytes show hollowing of matrix particles, with an increase in size and rounding and a decrease or loss of cristae. Many studies have shown that when treatment for NAFLD is effective, liver mitochondrial function also improves (Wang et al., 2012; Lotowska et al., 2014; Zhenhua, 2016). The membrane permeability of liver mitochondria can partly reflect their membrane function, and the ability of mitochondrial oxidative phosphorylation to produce ATP is reflective of mitochondrial respiratory function. Our preliminary experiments showed that Shenling Jianquan gastric granules (an additive formula of SLBZP) enhanced hepatic UCP2 expression in NAFLD mice (Yao et al., 2017). Metformin, commonly used in diabetes treatment, is also an activator of AMPK, which inhibits gluconeogenesis in the liver, promotes fatty acid oxidation and improves insulin sensitivity, thereby lowering blood glucose levels (Hawley et al., 2002; Liu et al., 2006). Metformin can treat NAFLD by modulating insulin resistance and improving hepatic steatosis, hepatocyte injury, liver serum biochemical indices and body weight (Idilman et al., 2008; Loomba et al., 2009). Our experimental results showed that SLBZP improved mitochondrial membrane permeability and the mitochondrial respiratory state of the liver cells of NAFLD mice, increased the mitochondrial OPR and ATP levels in the liver of NAFLD mice and regulated the UCP2/AMPK/IF1 signalling pathway, thereby affecting mitochondrial energy metabolism.

Our study investigated the relationship between intestinal homeostasis and hepatic energy metabolism based on interactions between the intestine and liver and provides a basis for interpreting the scientific meaning of the TCM theory of “treating the liver benefits the spleen”. To further clarify the molecular mechanism underlying the effect of SCFAs on mitochondrial energy metabolism, an HFD-induced cell model could be used to observe the effect of SCFAs on the UCP2/AMPK/IF1 signalling pathway proteins of mitochondrial energy metabolism in liver cells. Owing to limitations of this study, we lack further verification of the obtained findings; nevertheless, the results warrant continued exploration.

## 5. Conclusion

Our experimental results showed that SLBZP, a classic formula for strengthening the spleen, reduces lipid deposition in the liver of mice with HFD-induced NAFLD. SLBZP regulates the UCP2/AMPK/IF1 signalling pathway involved in hepatic mitochondrial energy metabolism through SCFAs produced by intestinal flora metabolism. It can improve the permeability of the liver mitochondrial membrane, respiratory status and oxidative phosphorylation efficiency and increase the ATP level in the liver.

## Data availability statement

The datasets presented in this study can be found in online repositories. The names of the repository/repositories and accession number(s) can be found in the article/Supplementary material.

## Ethics statement

The animal study was reviewed and approved by Yunnan College of Traditional Chinese Medicine Animal Experiment Ethics Review Committee.

## Author contributions

ZY and AS designed this study and wrote a manuscript. JG conducted data analysis and wrote a manuscript. BD and LH conducted experiments and data analysis. JG, YZ, YH, and XF participated in some experiments and plotted them. All authors contributed to the article and approved the submitted version.

## Funding

This study was funded by the National Natural Science Foundation of China (no. 81860812), Yunnan Provincial Department of Science and Technology Joint Special Project of Traditional Chinese Medicine—Key Project (no. 202001AZ070001-005).

## Conflict of interest

The authors declare that the research was conducted in the absence of any commercial or financial relationships that could be construed as a potential conflict of interest.

## Publisher’s note

All claims expressed in this article are solely those of the authors and do not necessarily represent those of their affiliated organizations, or those of the publisher, the editors and the reviewers. Any product that may be evaluated in this article, or claim that may be made by its manufacturer, is not guaranteed or endorsed by the publisher.
