# Supplementary material for: Effects of Shenling Baizhu powder on intestinal microflora metabolites and liver mitochondrial energy metabolism in nonalcoholic fatty liver mice
Source: Front Microbiol. 2023 Jul 18;14:1147067. doi: 10.3389/fmicb.2023.1147067 (PMC10394096; doi:10.3389/fmicb.2023.1147067)

F:\中药非靶\...\NEG\ZY 09/28/22 02:33:36

Ginsenoside Rg2-neg-Secondary mass spectrum

ZY #2511 RT: 4.65 AV: 1 NL: 1.65E5

F: FTMS - p ESI d Full ms2 829.4968@hcd32.00 [86.6852-866.8517]

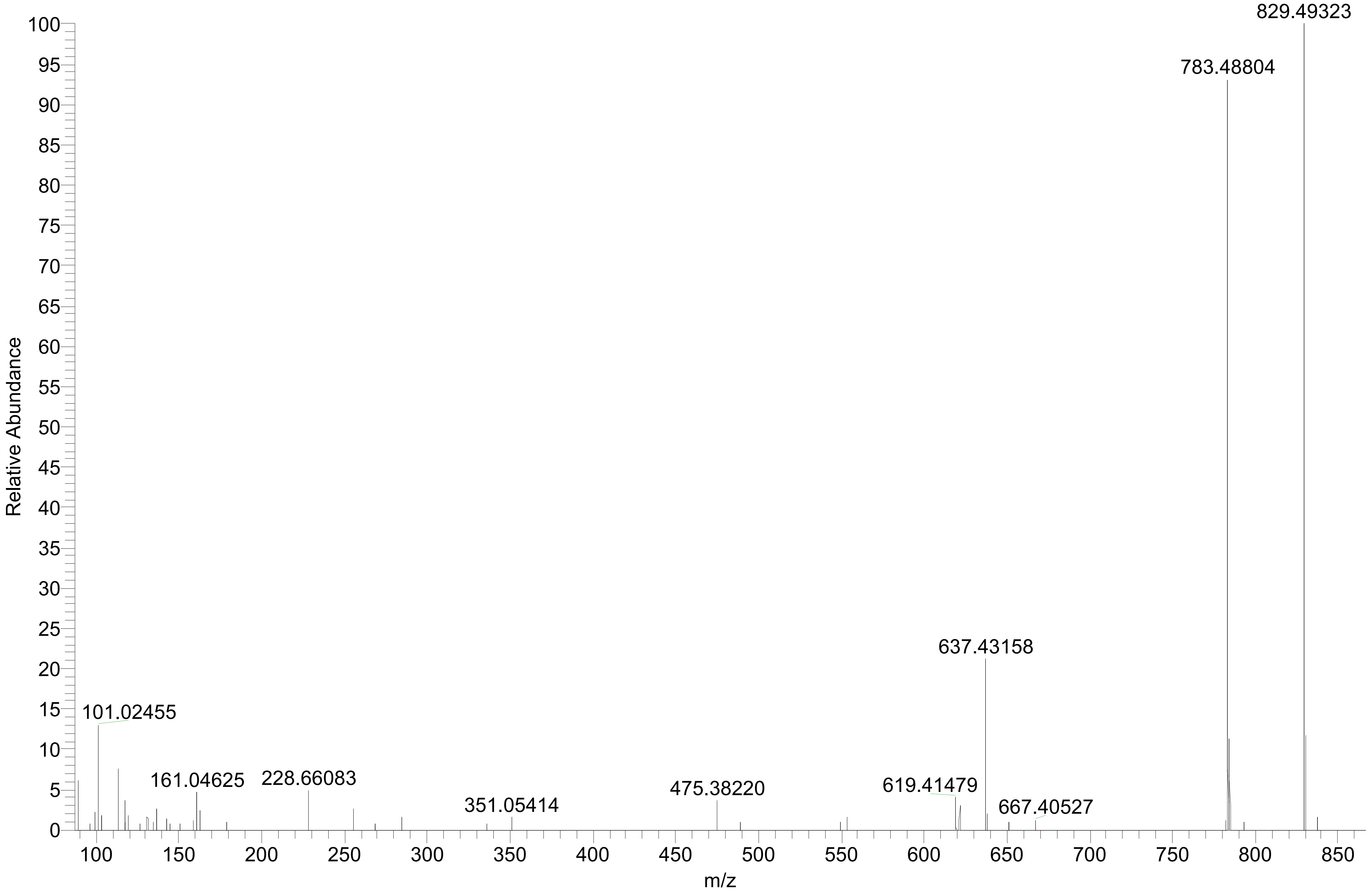

F:\中药非靶\...\NEG\ZY Ginsenoside Rg3(S-FORM)-neg 09/28/22 02:33:36

ZY #4464 RT: 8.00 AV: 1 NL: 1.88E6
F: FTMS - p ESI d Full ms2 783.4910@hcd32.00 [81.9926-819.9258]

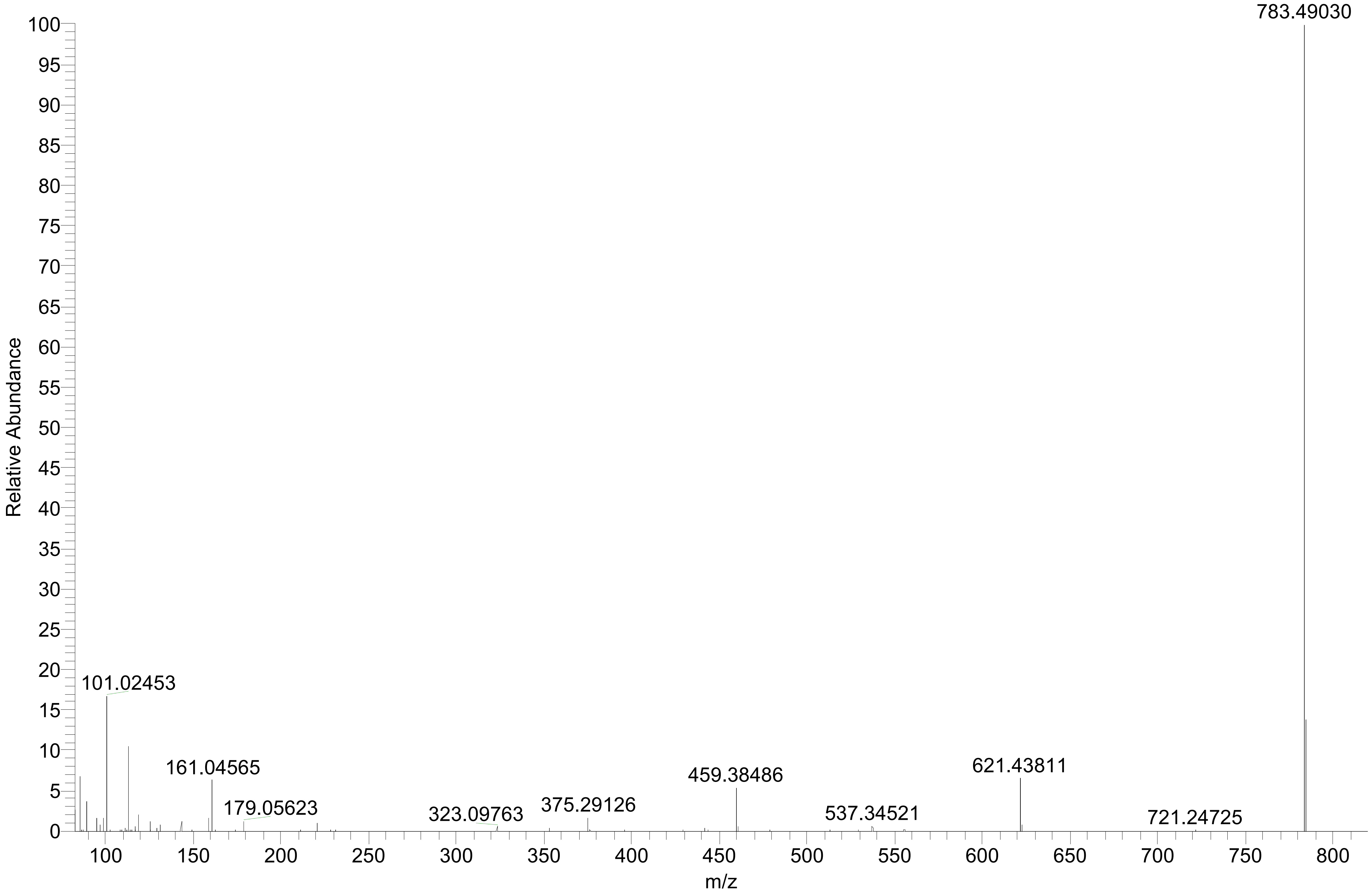

F:\中药非靶\...\POS\ZY Ginsenoside Rg5-pos 09/27/22 21:29:09

ZY #2892 RT: 5.09 AV: 1 NL: 3.54E4
F: FTMS + p ESI d Full ms2 767.4938@hcd32.00 [80.3609-803.6087]

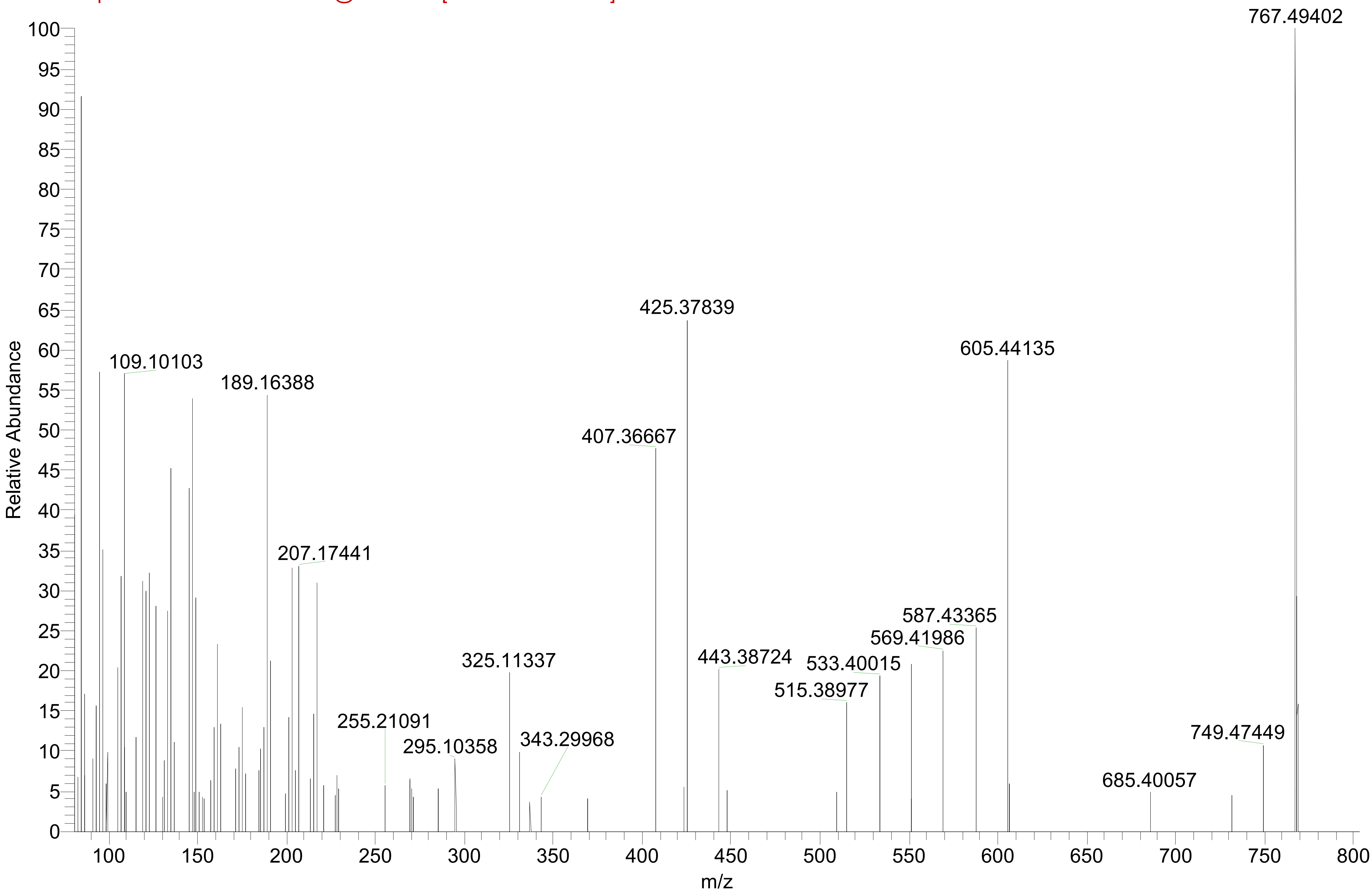

F:\中药非靶\...\NEG\ZY Ginsenoside Ro-neg 09/28/22 02:33:36

ZY #2746 RT: 5.06 AV: 1 NL: 1.35E6

F: FTMS - p ESI d Full ms2 955.4543@hcd32.00 [99.5328-995.3284]

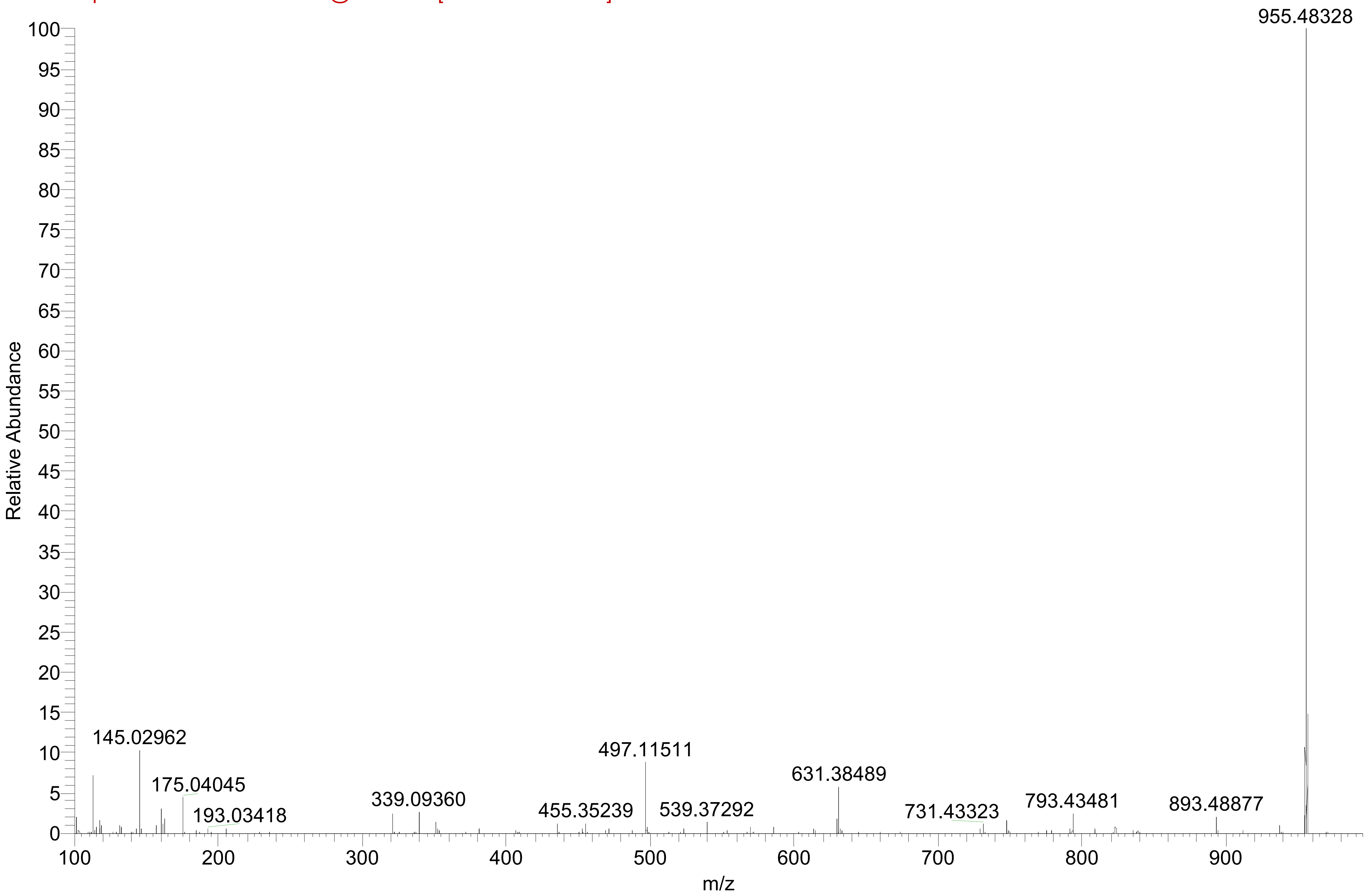

F:\中药非靶\...\NEG\ZY Licoricesaponin G2-neg 09/28/22 02:33:36

ZY #2664 RT: 4.92 AV: 1 NL: 1.59E8
F: FTMS - p ESI d Full ms2 837.3917@hcd32.00 [87.4904-874.9045]

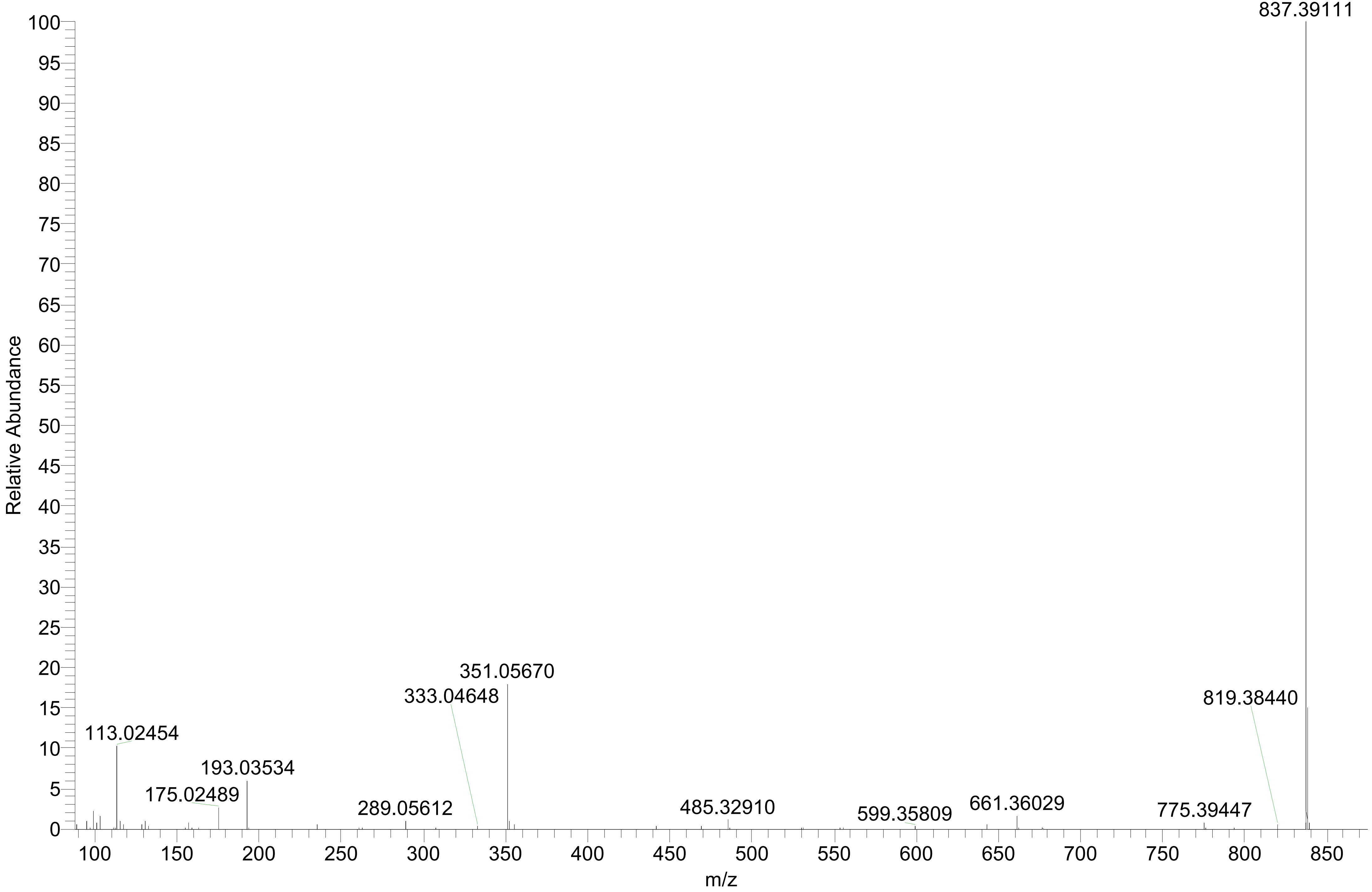

F:\中药非靶\...\POS\ZY Atractylenolide III-pos-二级 09/27/22 21:29:09

ZY #4017 RT: 6.80 AV: 1 NL: 2.98E6
F: FTMS + p ESI d Full ms2 249.1481@hcd32.00 [50.0000-274.8960]

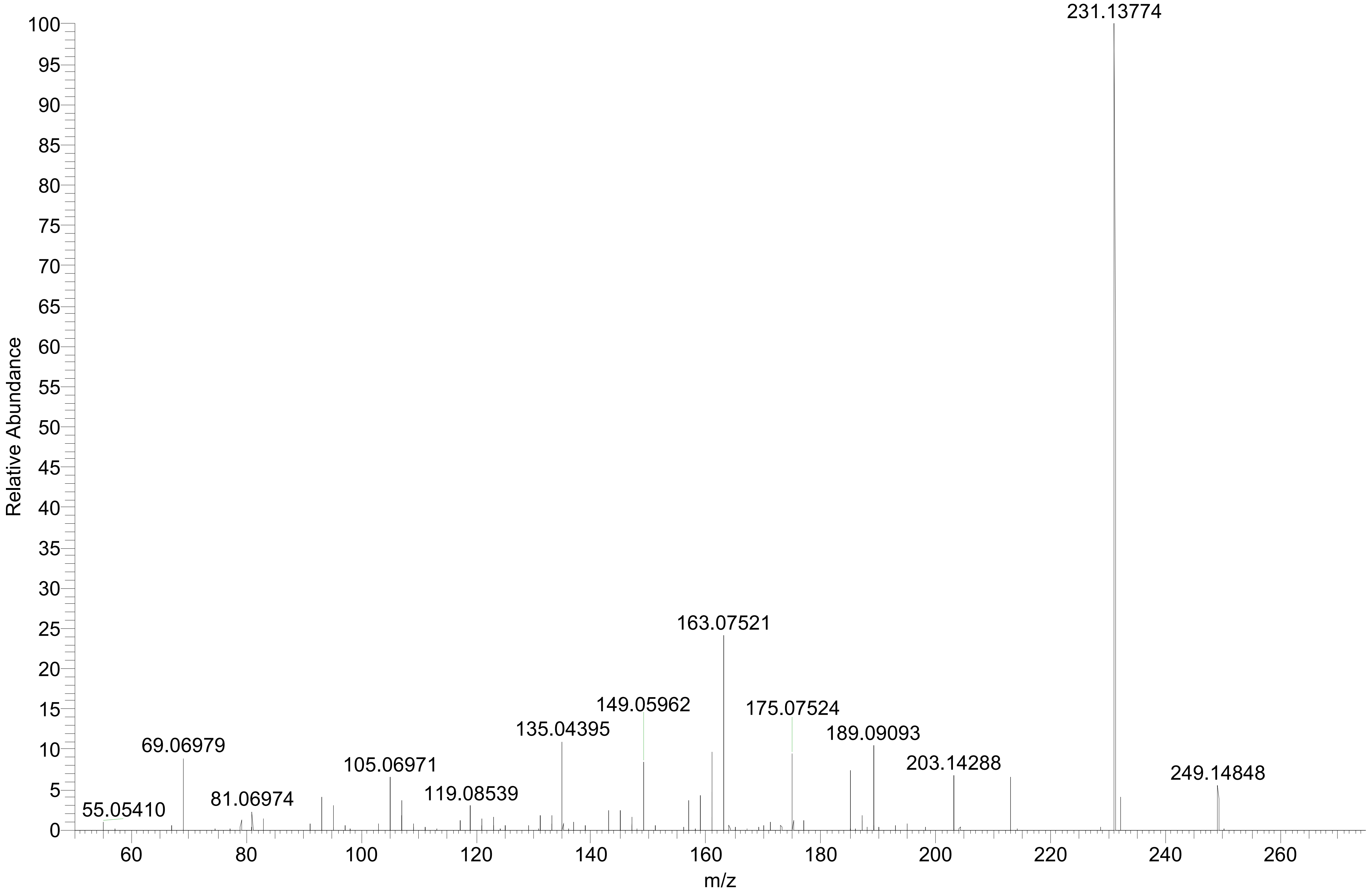

F:\中药非靶\...\POS\ZY quercetin-pos-二级 09/27/22 21:29:09

ZY #837 RT: 1.56 AV: 1 NL: 8.33E5

F: FTMS + p ESI d Full ms2 303.0860@hcd32.00 [50.0000-329.9128]

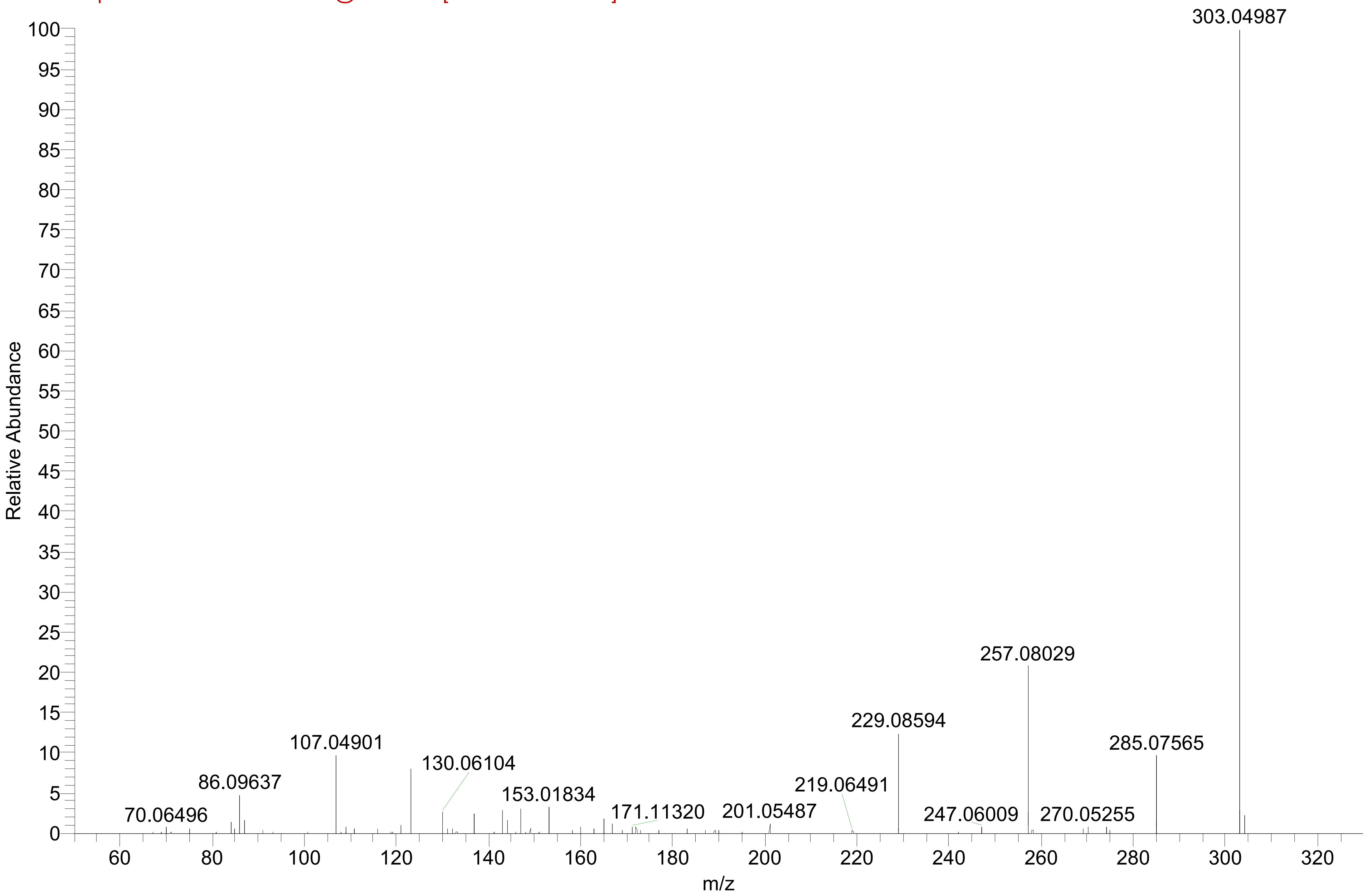

ZY #806 RT: 1.58 AV: 1 NL: 5.62E5
F: FTMS - p ESI d Full ms2 463.1255@hcd32.00 [50.0000-493.1530]

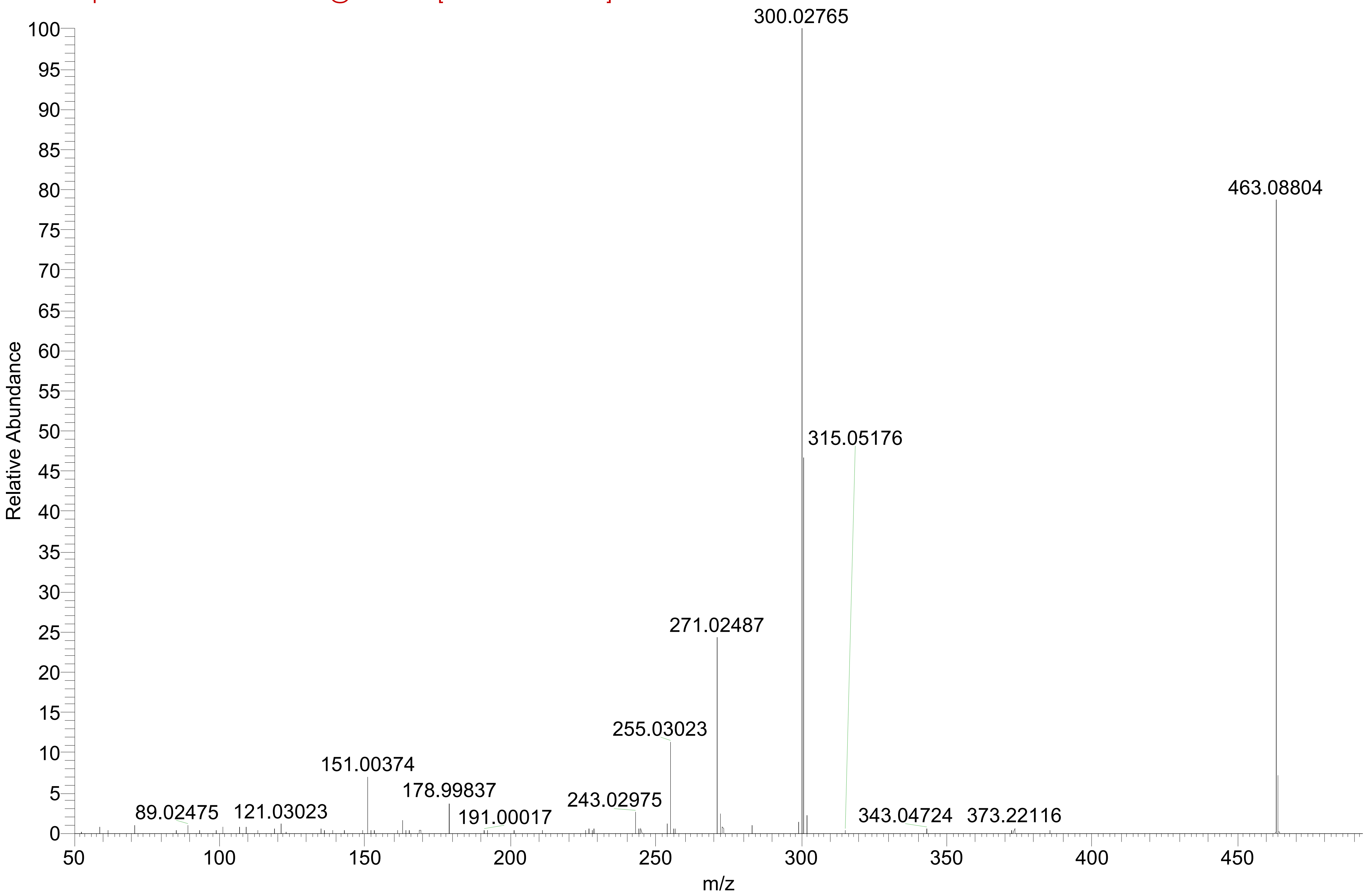

F:\中药非靶\...\POS\ZY Quercetin-3-O-galactoside-pos-二级 09/27/22 21:29:09

ZY #847 RT: 1.58 AV: 1 NL: 6.62E5
F: FTMS + p ESI d Full ms2 465.1023@hcd32.00 [50.0000-495.1693]

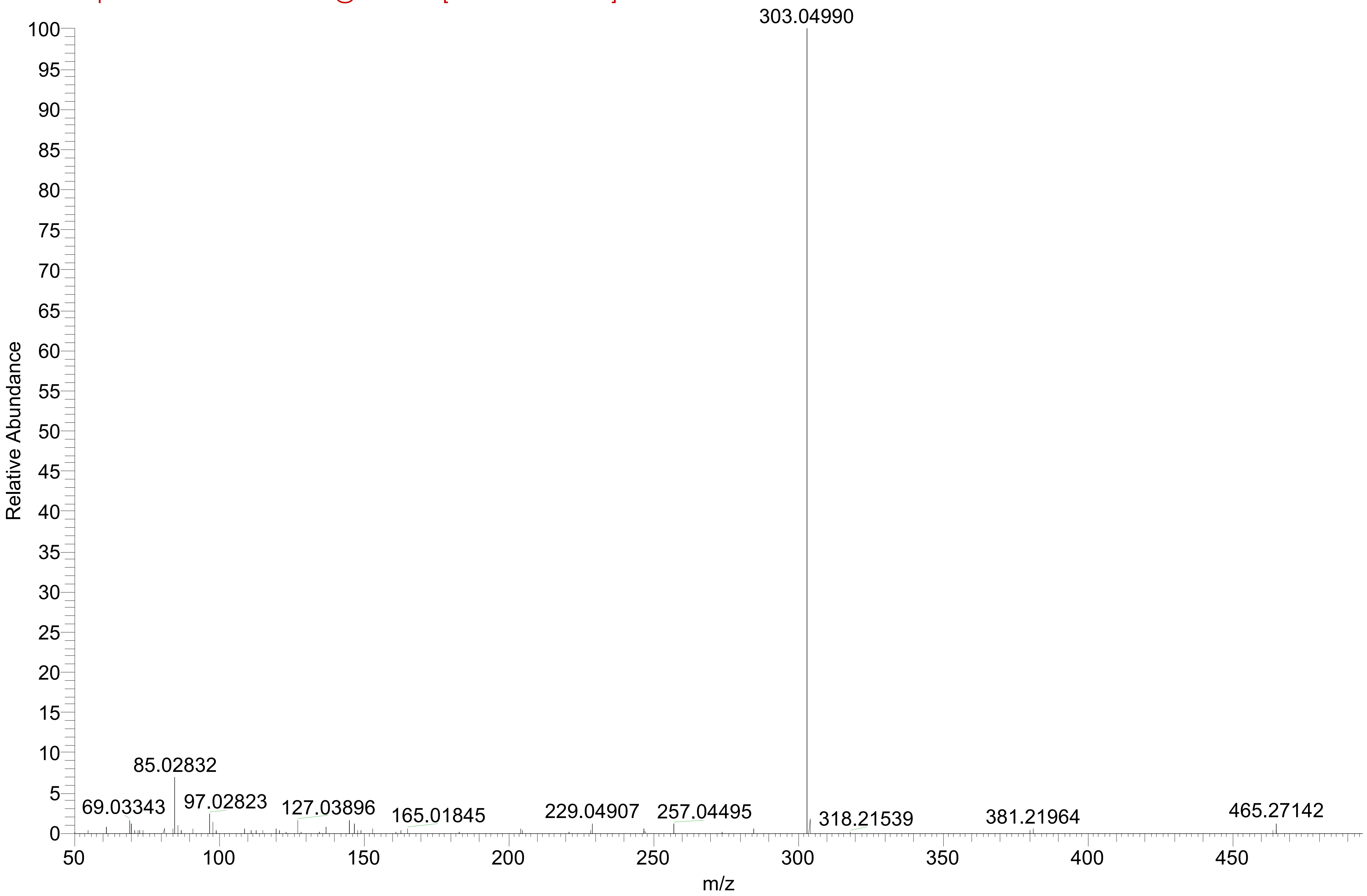

Supplement: Supplementary file 3 [file Data_Sheet_1.PDF]
