# Supplementary material for: Effects of Shenling Baizhu powder on intestinal microflora metabolites and liver mitochondrial energy metabolism in nonalcoholic fatty liver mice
Source: Front Microbiol. 2023 Jul 18;14:1147067. doi: 10.3389/fmicb.2023.1147067 (PMC10394096; doi:10.3389/fmicb.2023.1147067)

F:\中药非靶\...\NEG\ZY Ginsenoside Rg2-neg-EIC 09/28/22 02:33:36

RT: 0.00 - 16.00

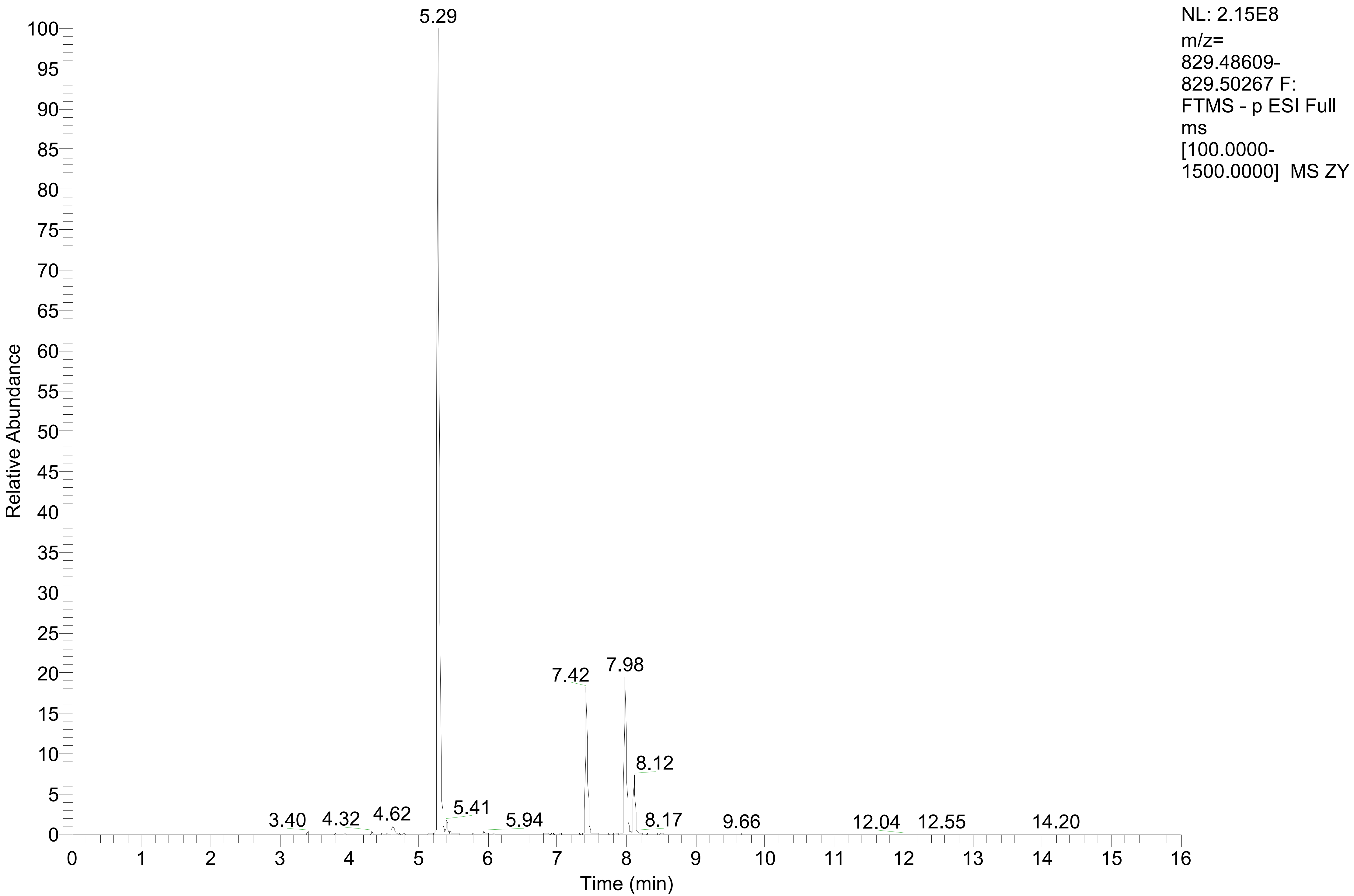

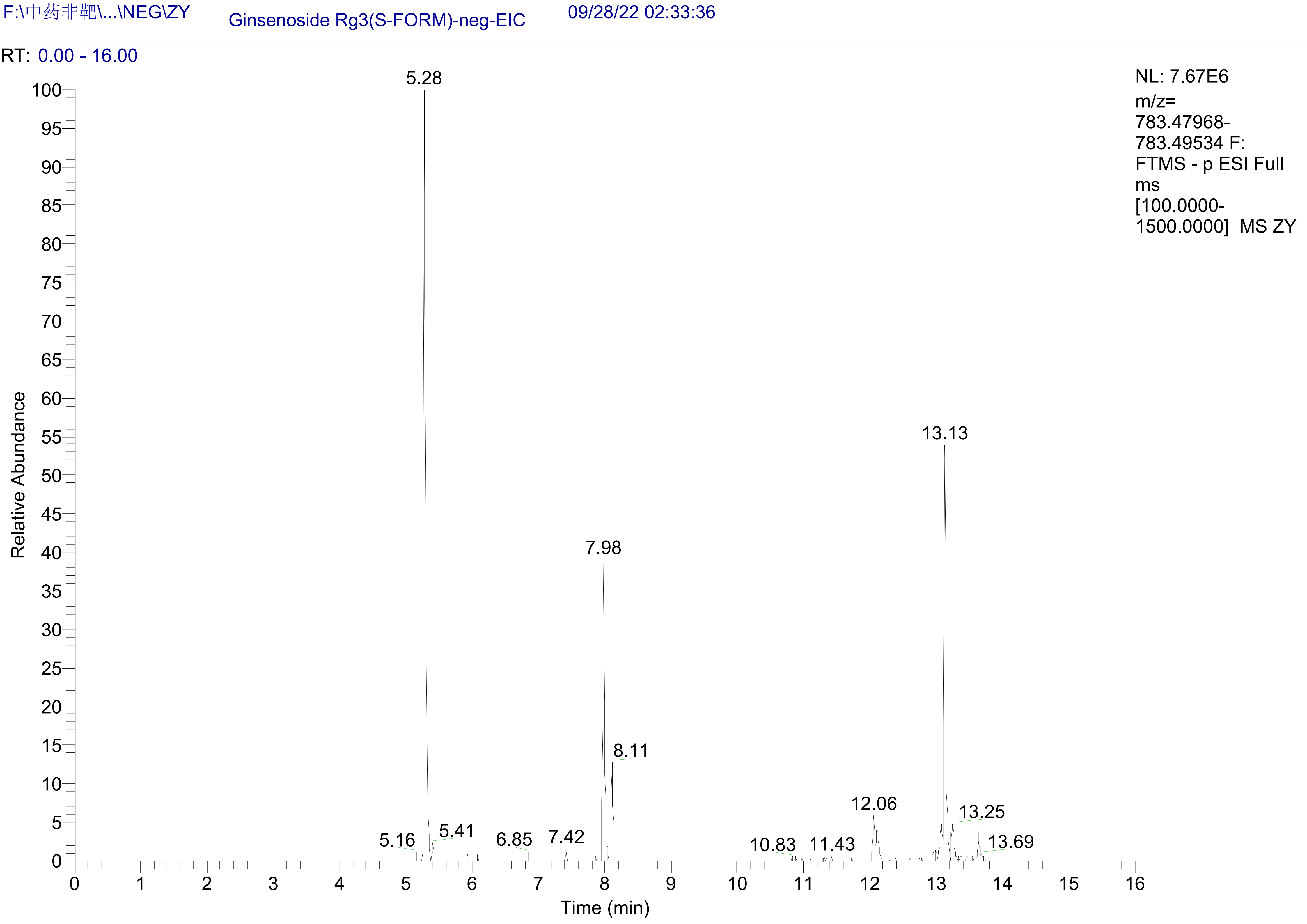
F:\中药非靶\...\NEG\ZY
Ginsenoside Rg3(S-FORM)-neg-EIC
09/28/22 02:33:36
RT: 0.00 - 16.00
NL: 7.67E6
m/z= 783.47968-783.49534 F: FTMS - p ESI Full ms [100.0000-1500.0000] MS ZY
Relative Abundance
Time (min)
5.28
13.13
7.98
8.11
12.06
13.25
5.16
5.41
6.85
7.42
10.83
11.43
13.69

F:\中药非靶\...\POS\ZY Ginsenoside Rg5-pos-EIC 09/27/22 21:29:09

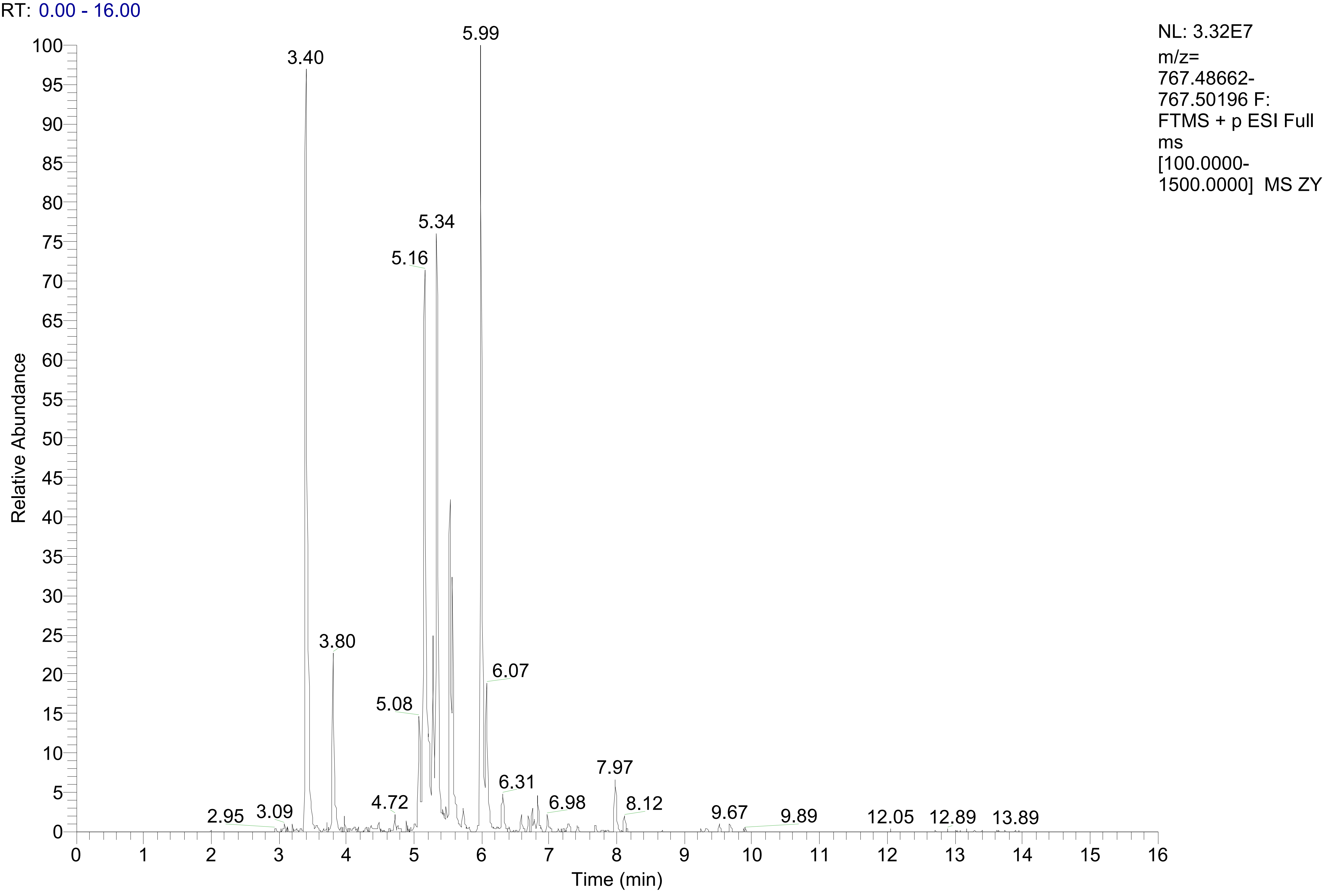

F:\中药非靶\...\NEG\ZY Ginsenoside Ro-neg-EIC 09/28/22 02:33:36

RT: 0.00 - 16.00

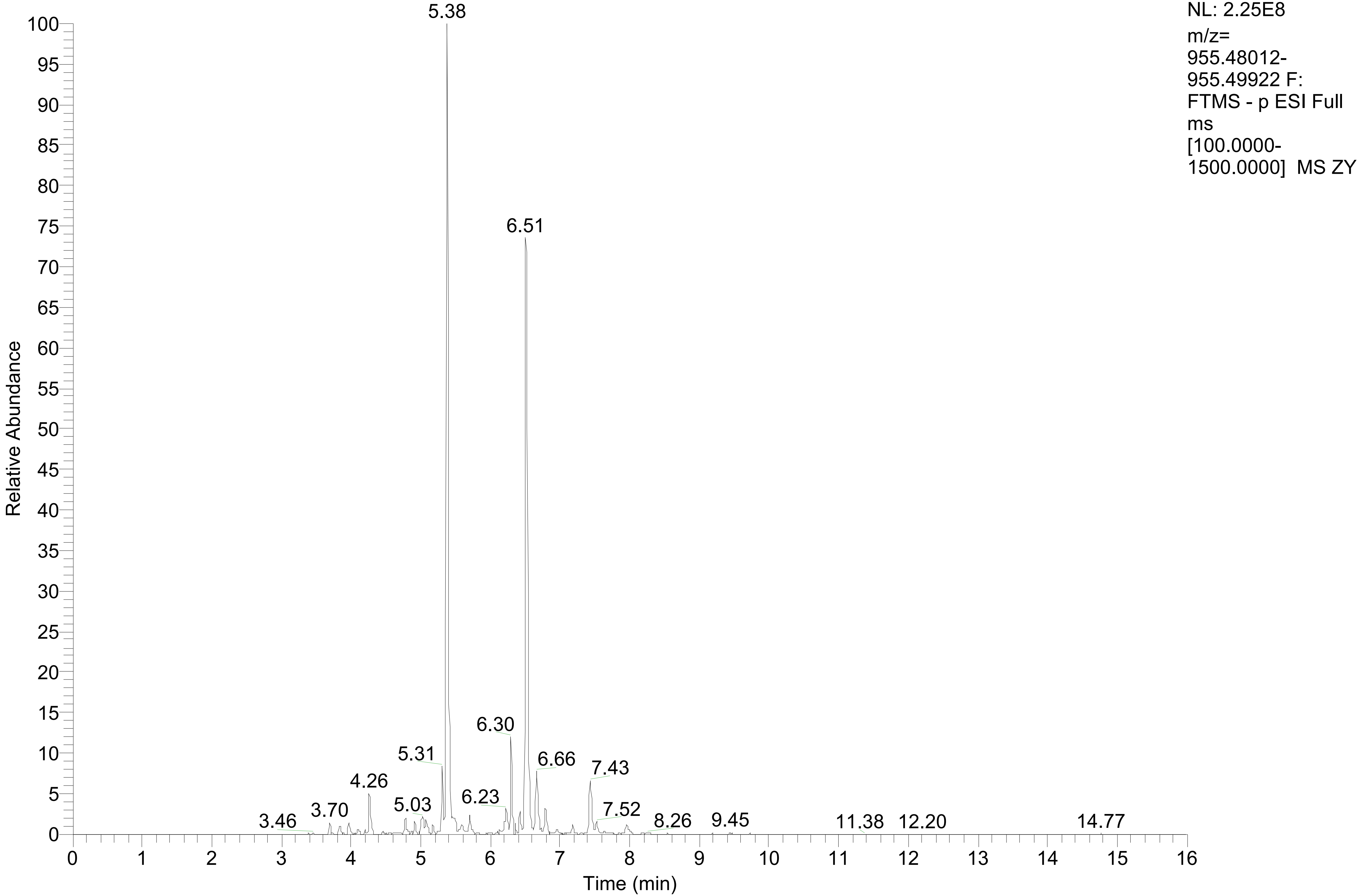

F:\中药非靶\...\NEG\ZY   Licoricesaponin G2-neg-EIC   09/28/22 02:33:36

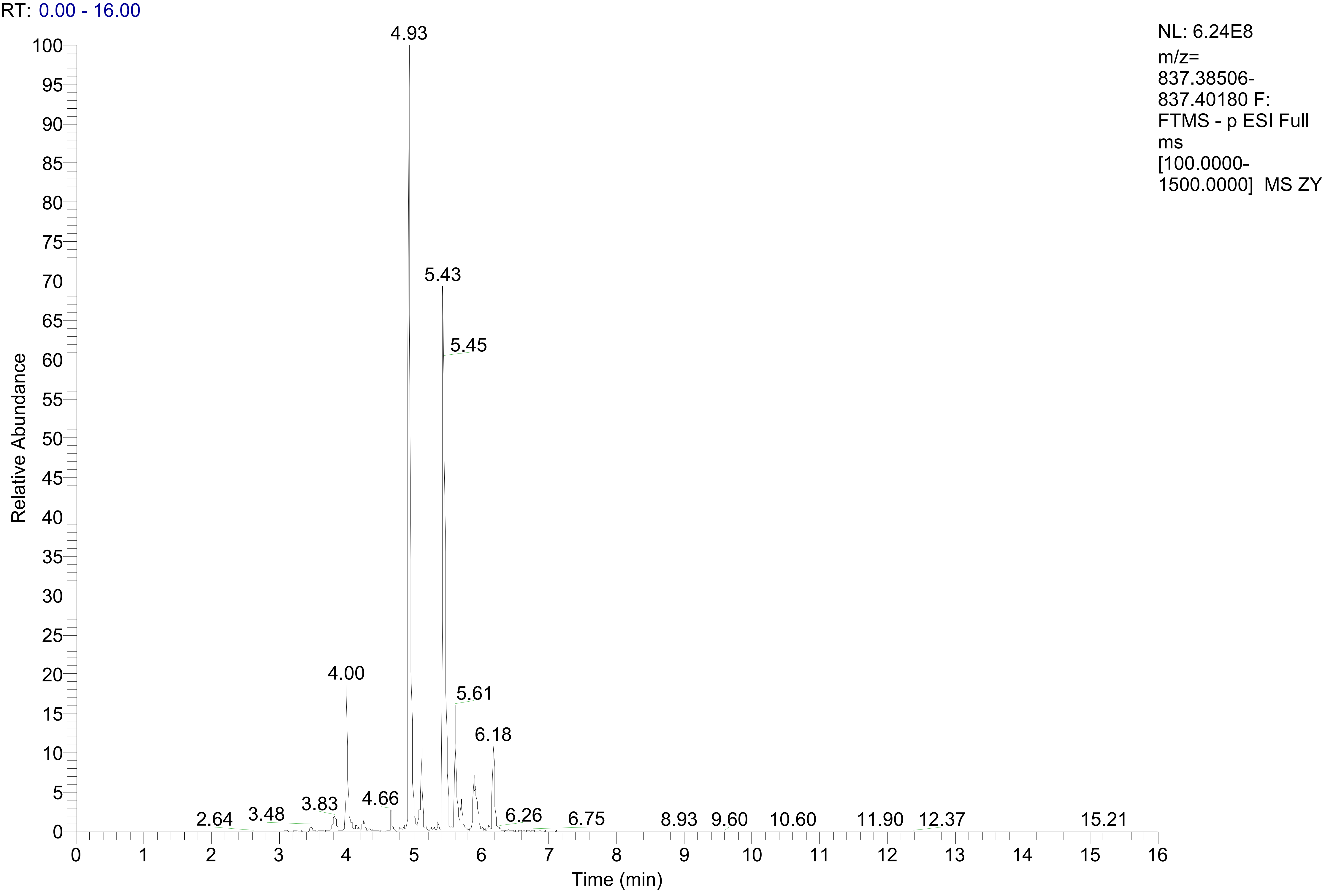

F:\中药非靶\...\POS\ZY Atractylenolide III-pos-EIC 09/27/22 21:29:09

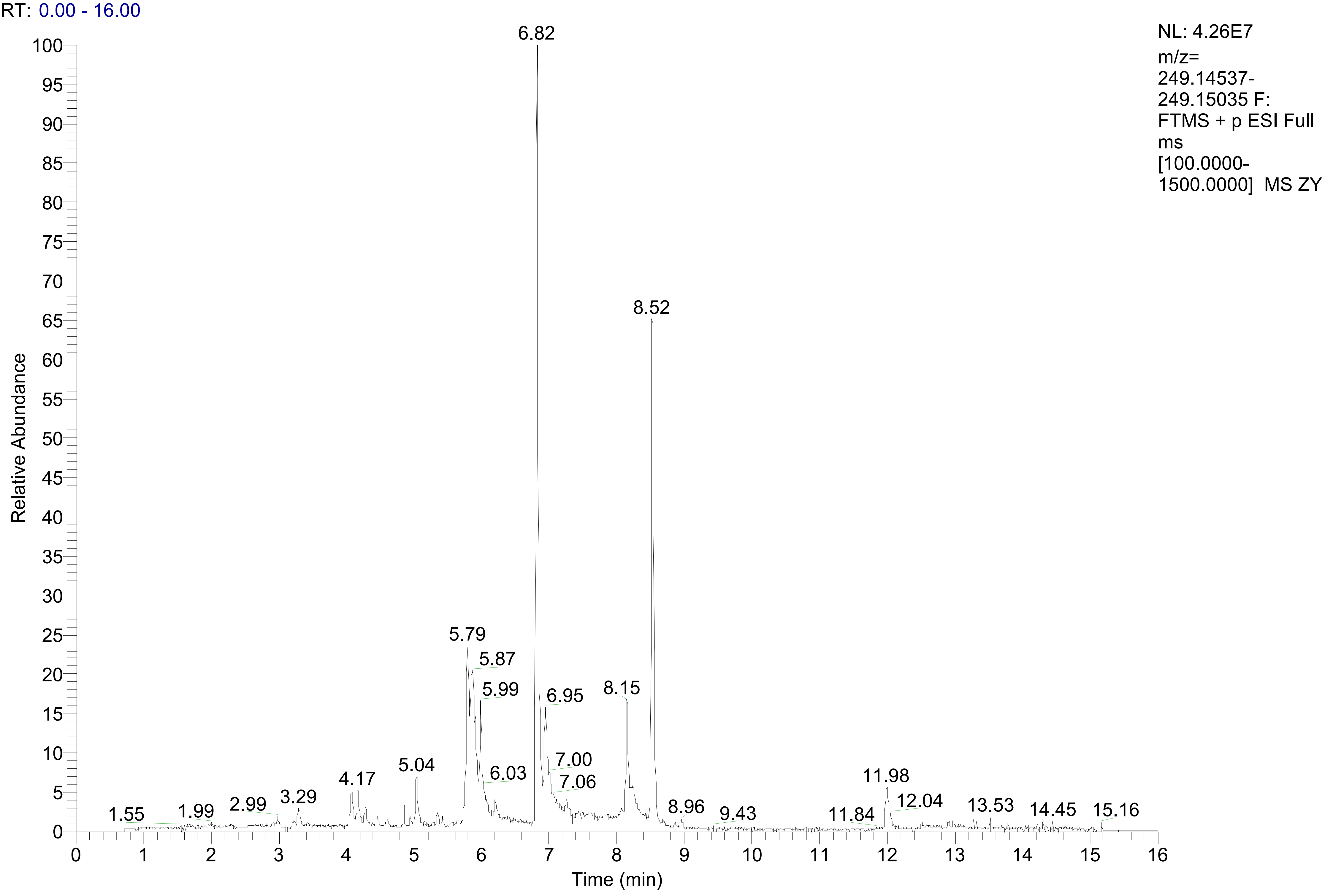

F:\中药非靶\...\POS\ZY quercetin-pos-EIC 09/27/22 21:29:09

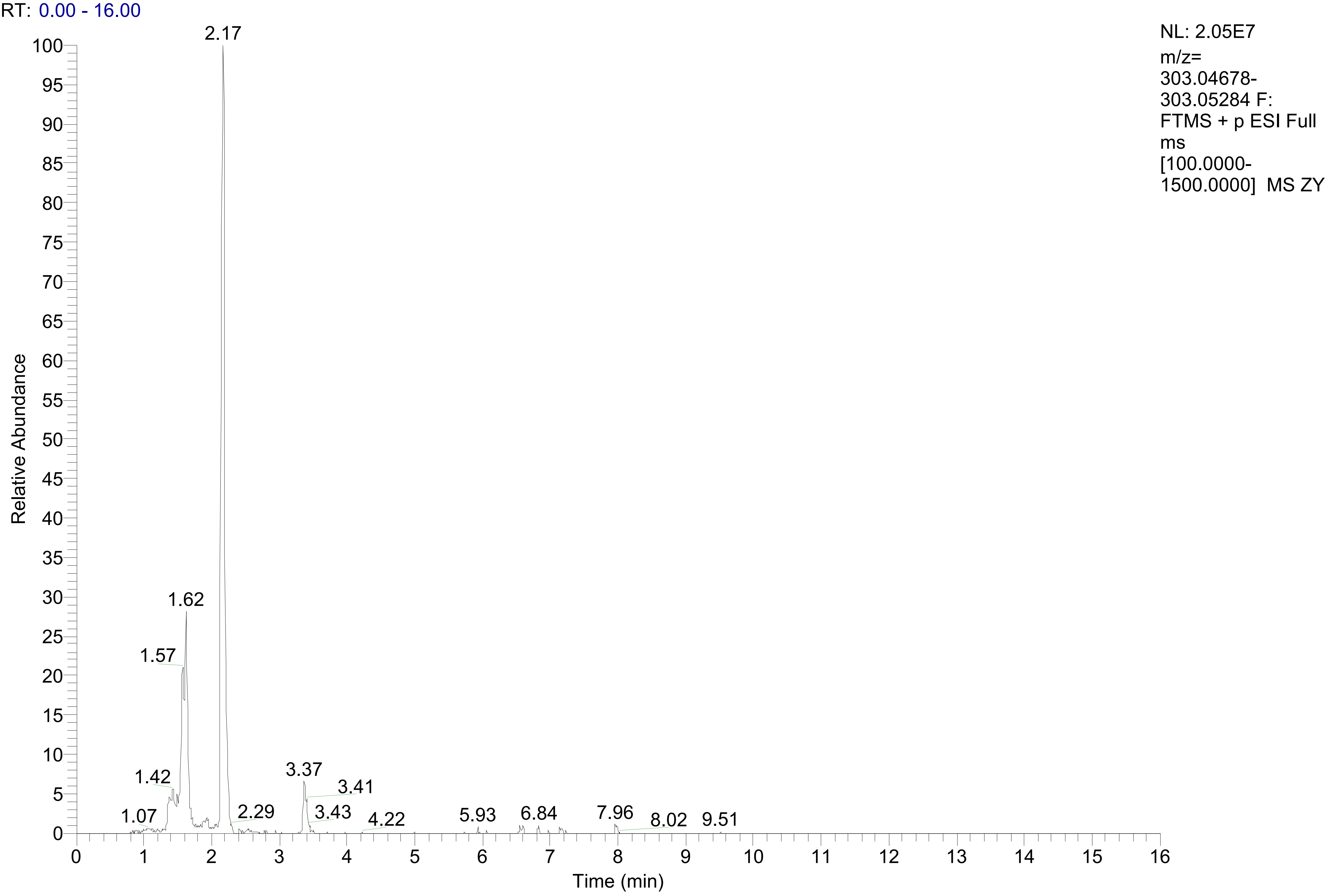

F:\中药非靶\...\NEG\ZY Quercetin-3-O-galactoside-neg-EIC 09/28/22 02:33:36

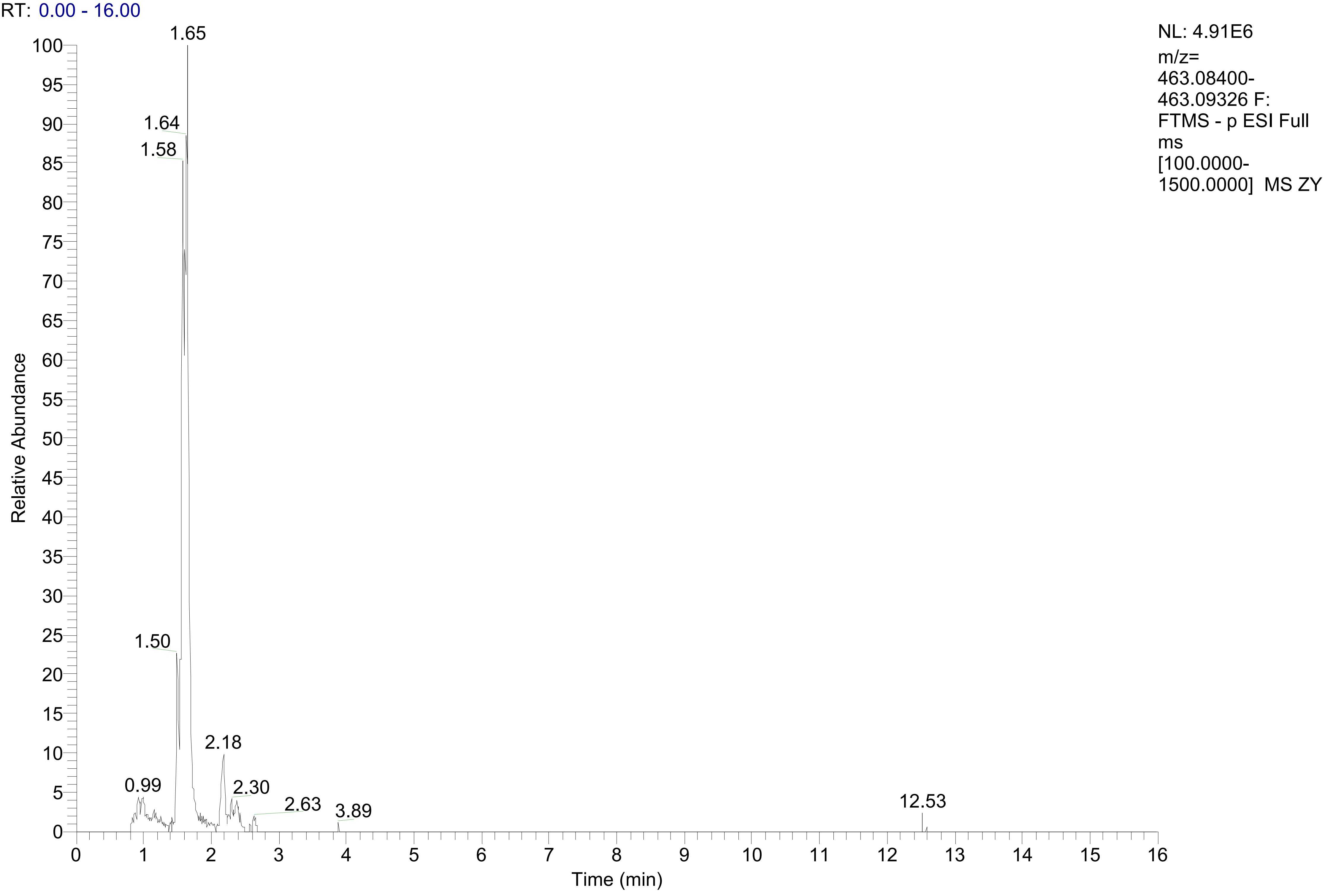

F:\中药非靶\...\POS\ZY Quercetin-3-O-galactoside-pos-EIC 09/27/22 21:29:09

RT: 0.00 - 16.00

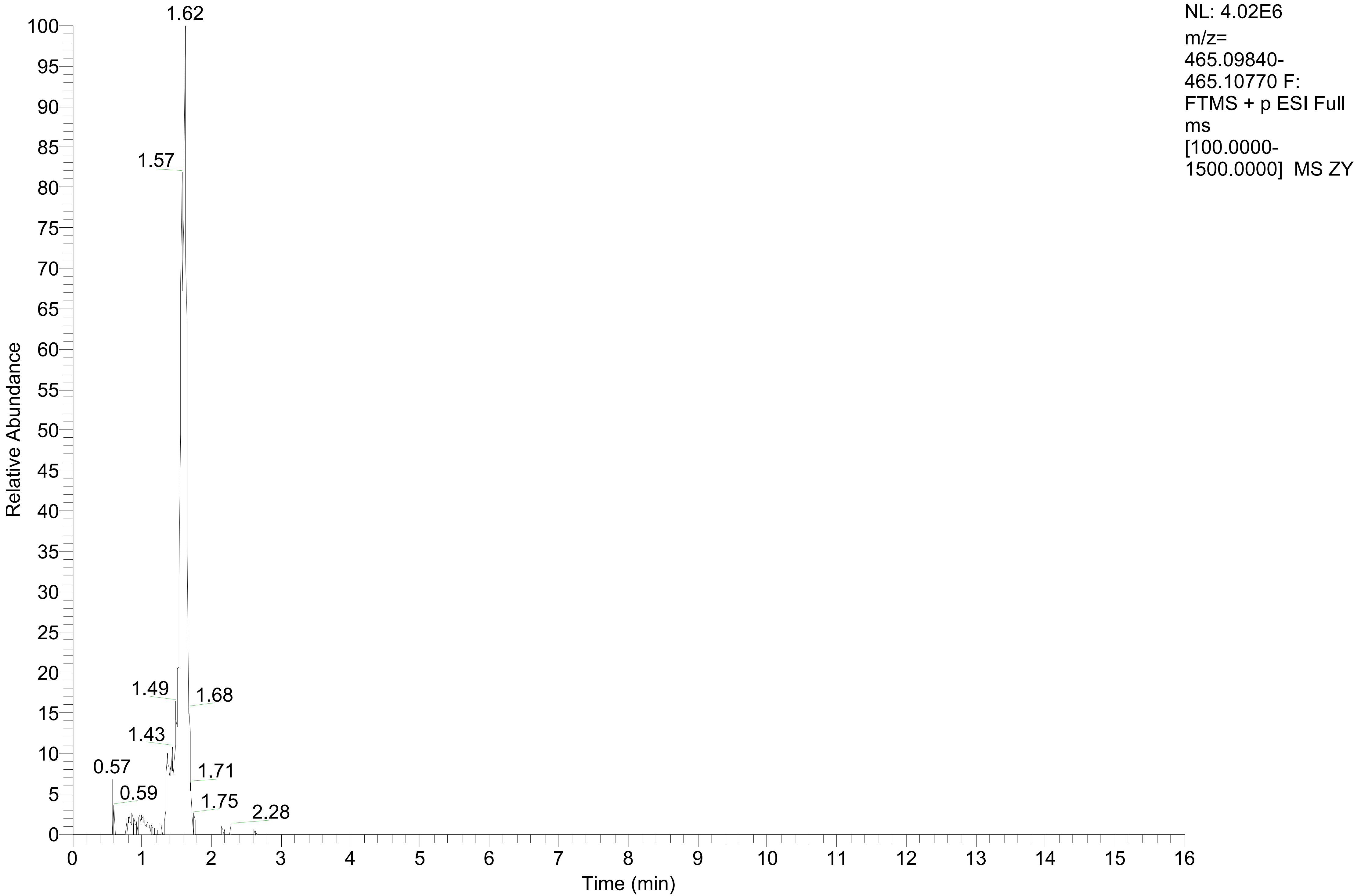

Supplement: Supplementary file 4 [file Data_Sheet_2.PDF]
