# Supplementary material for: Effects of Shenling Baizhu powder on intestinal microflora metabolites and liver mitochondrial energy metabolism in nonalcoholic fatty liver mice
Source: Front Microbiol. 2023 Jul 18;14:1147067. doi: 10.3389/fmicb.2023.1147067 (PMC10394096; doi:10.3389/fmicb.2023.1147067)
Supplement: Supplementary file 5 [file Data_Sheet_3.pdf]

Original data upload

HE staining diagram

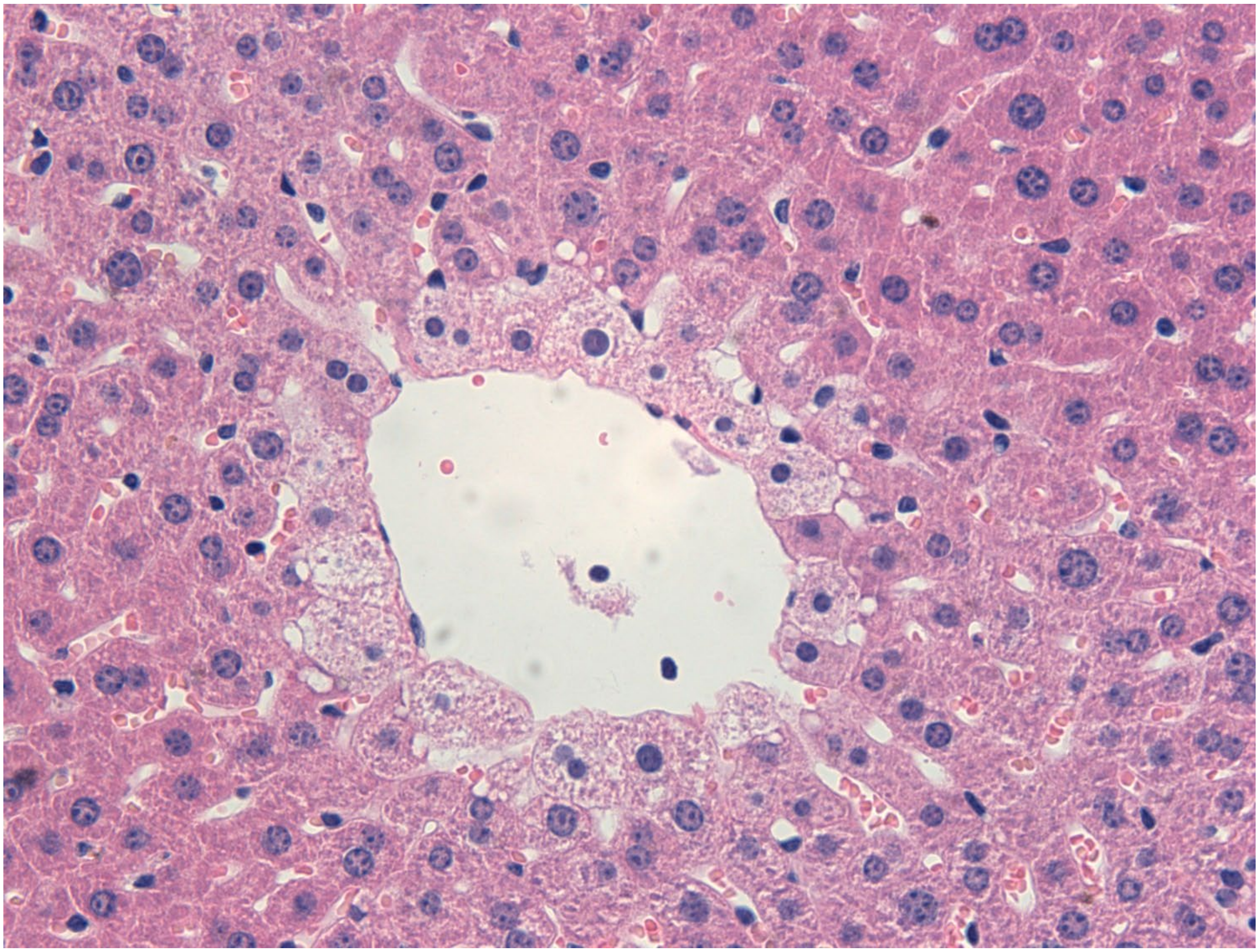

Normal group

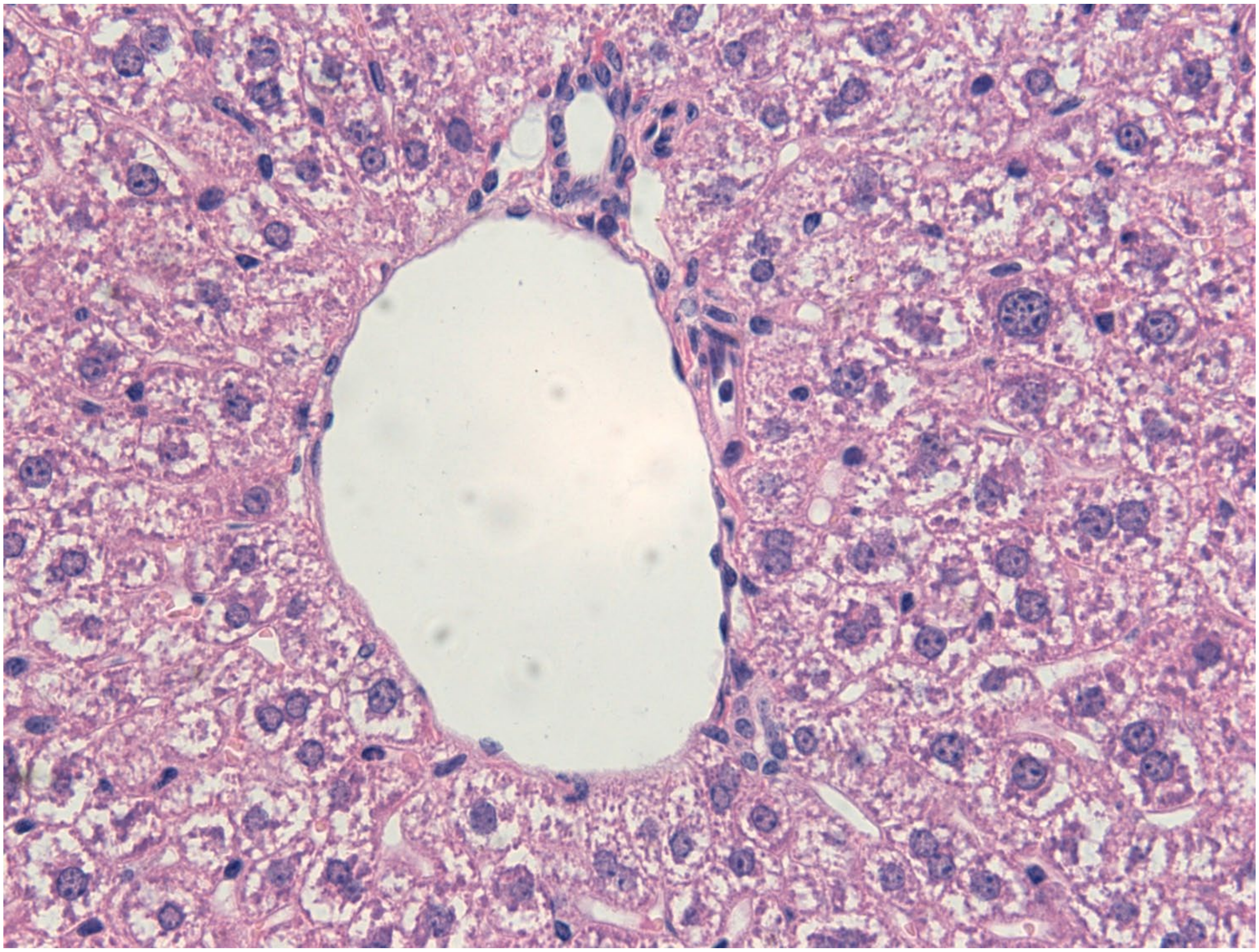

Model group

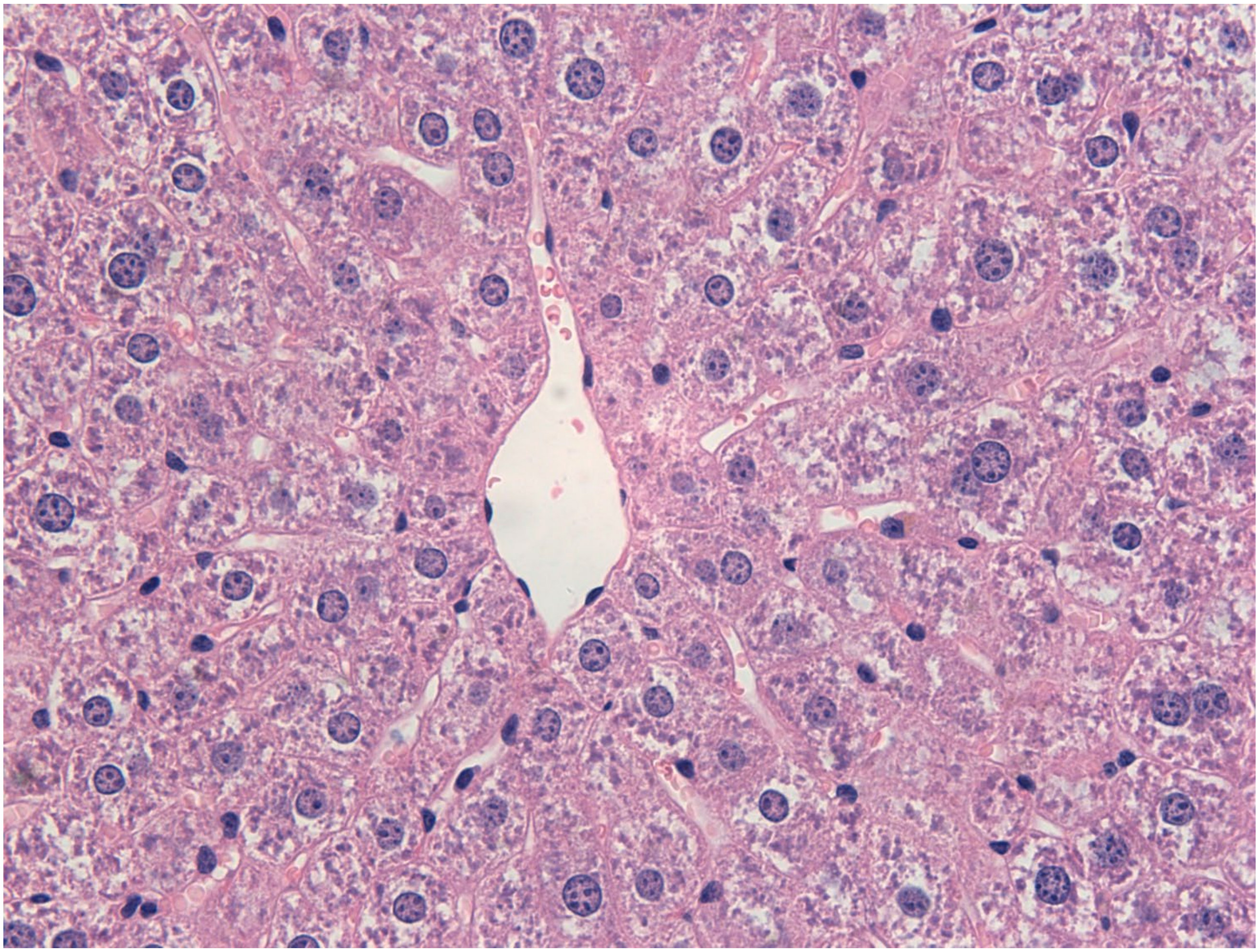

SLBZP Low-dose group

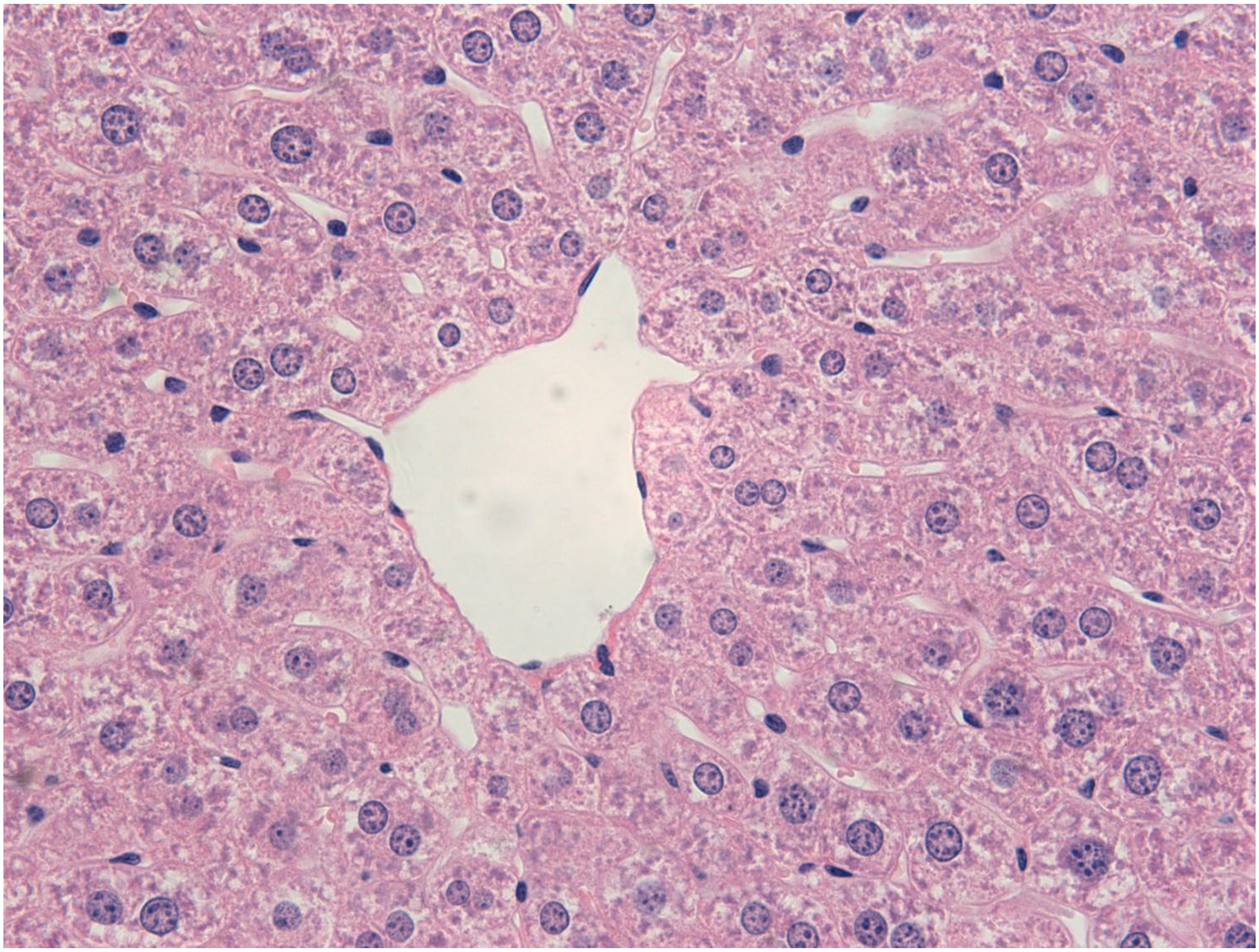

SLBZP Middle-dose group

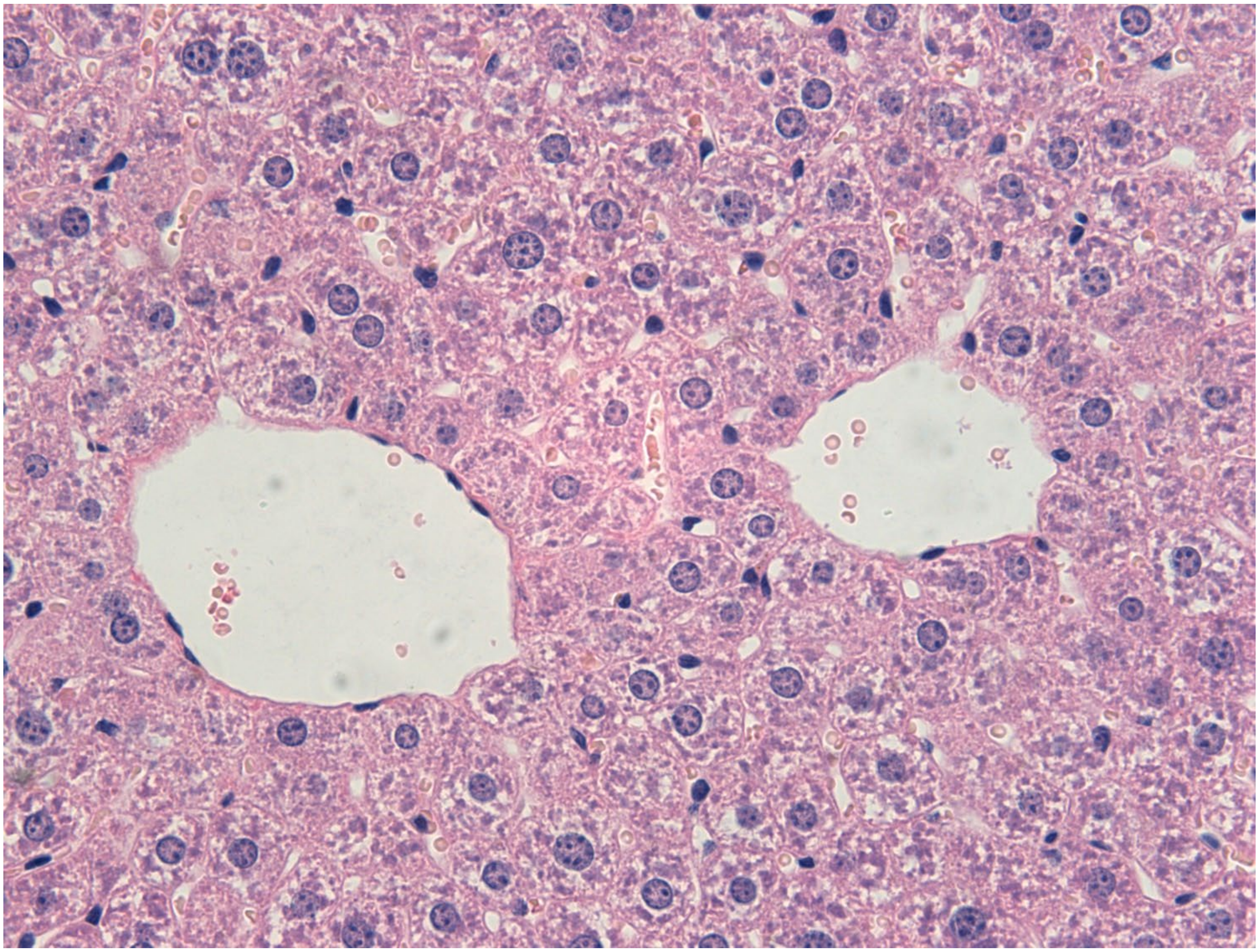

SLBZP High-dose group

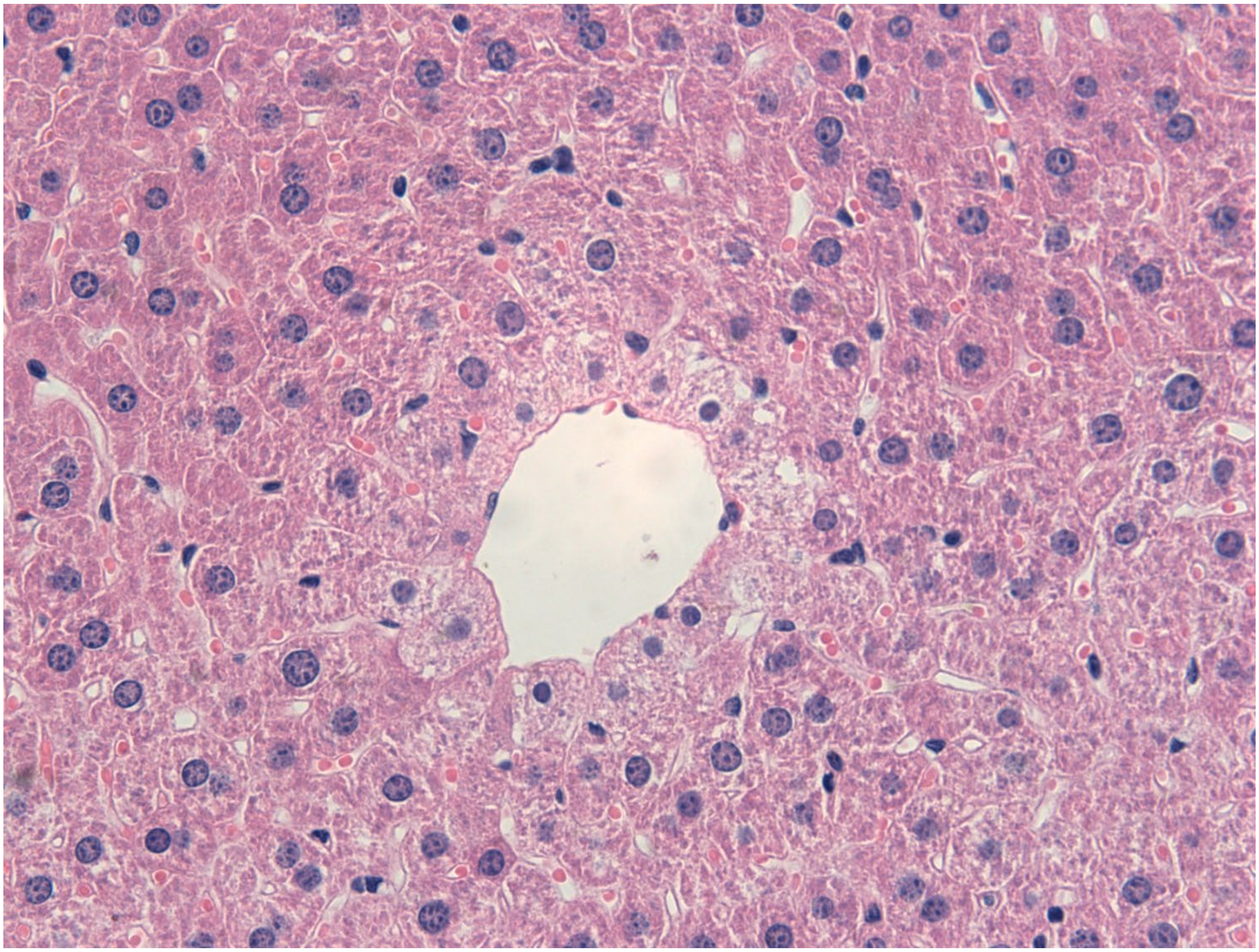

Metformin group

Oil red O staining diagram

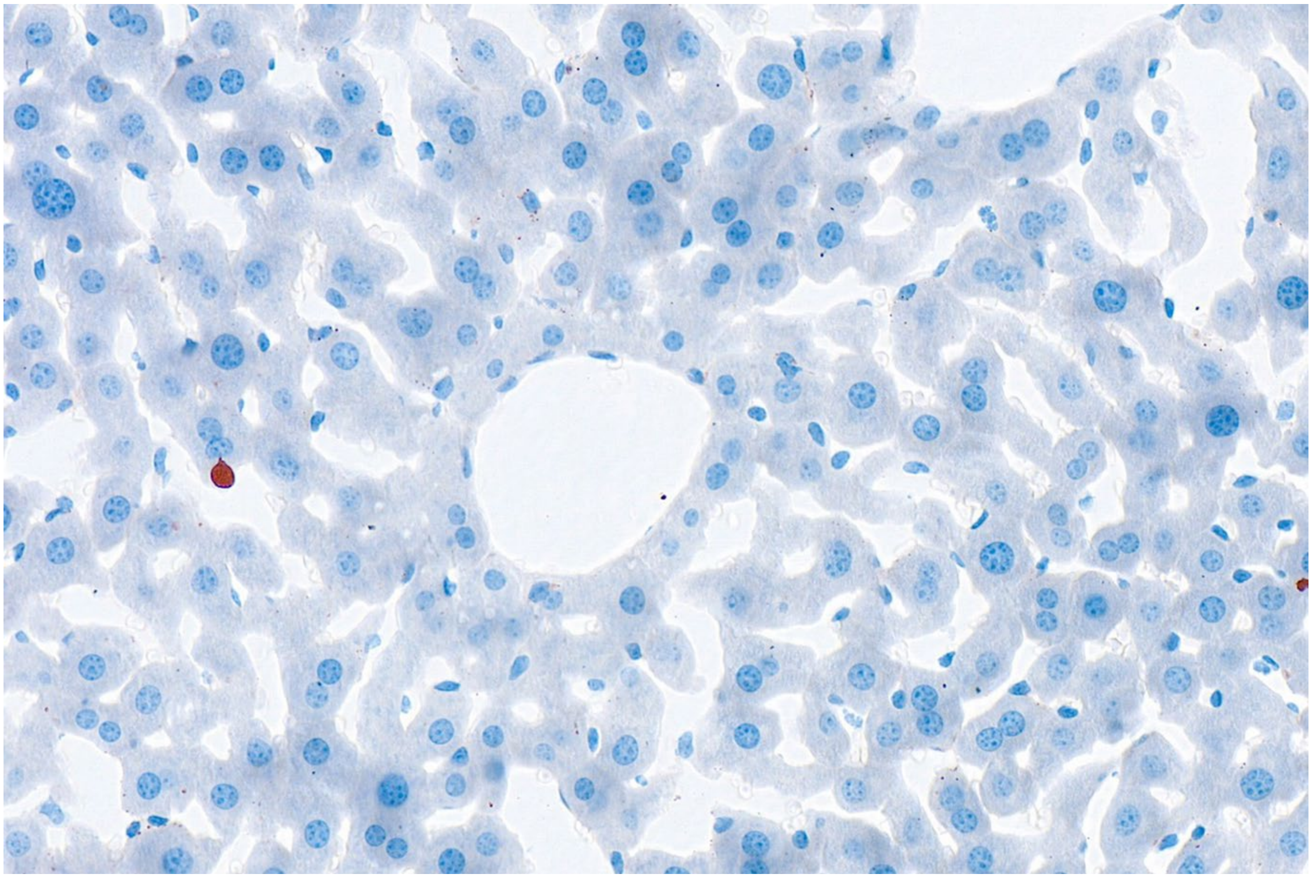

Normal group

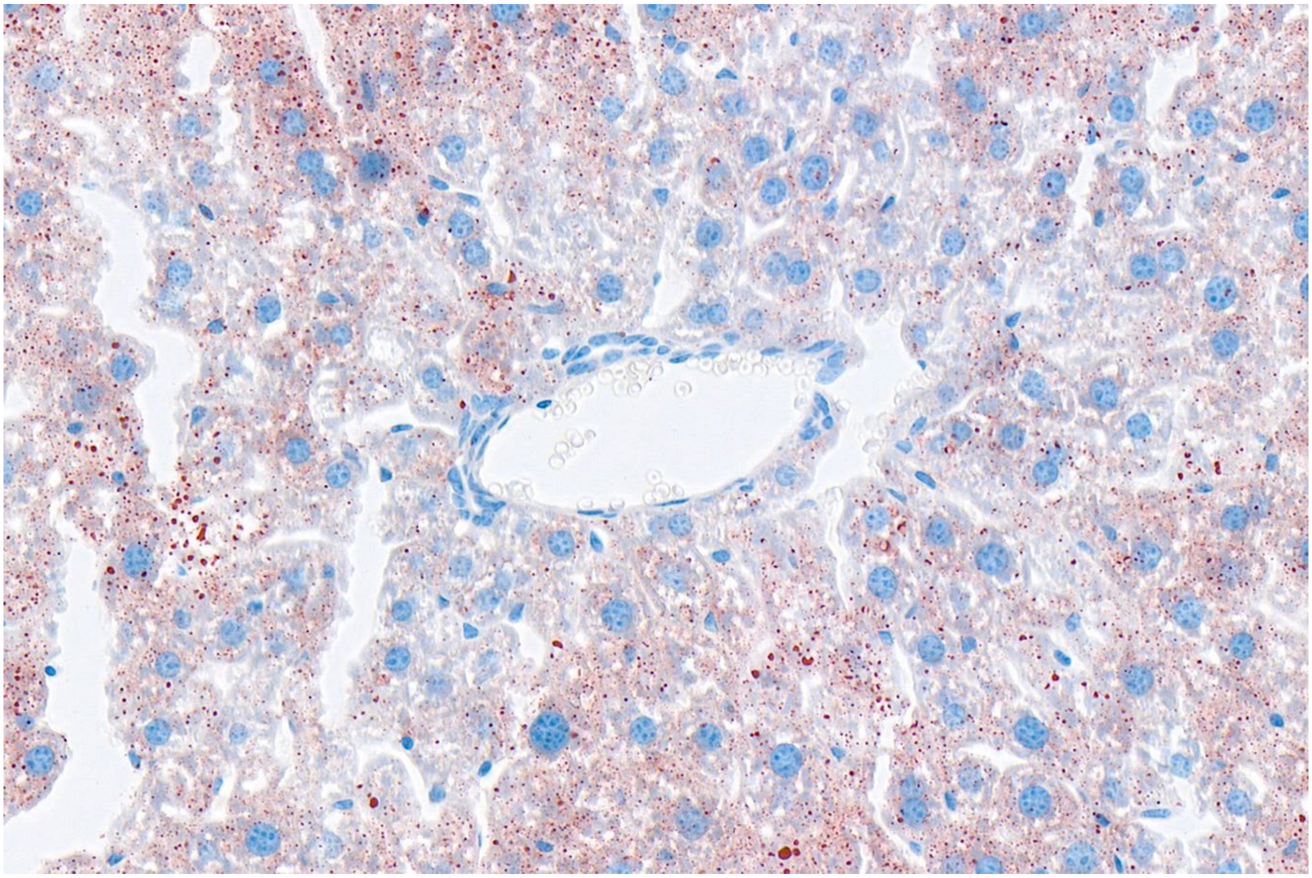

Model group

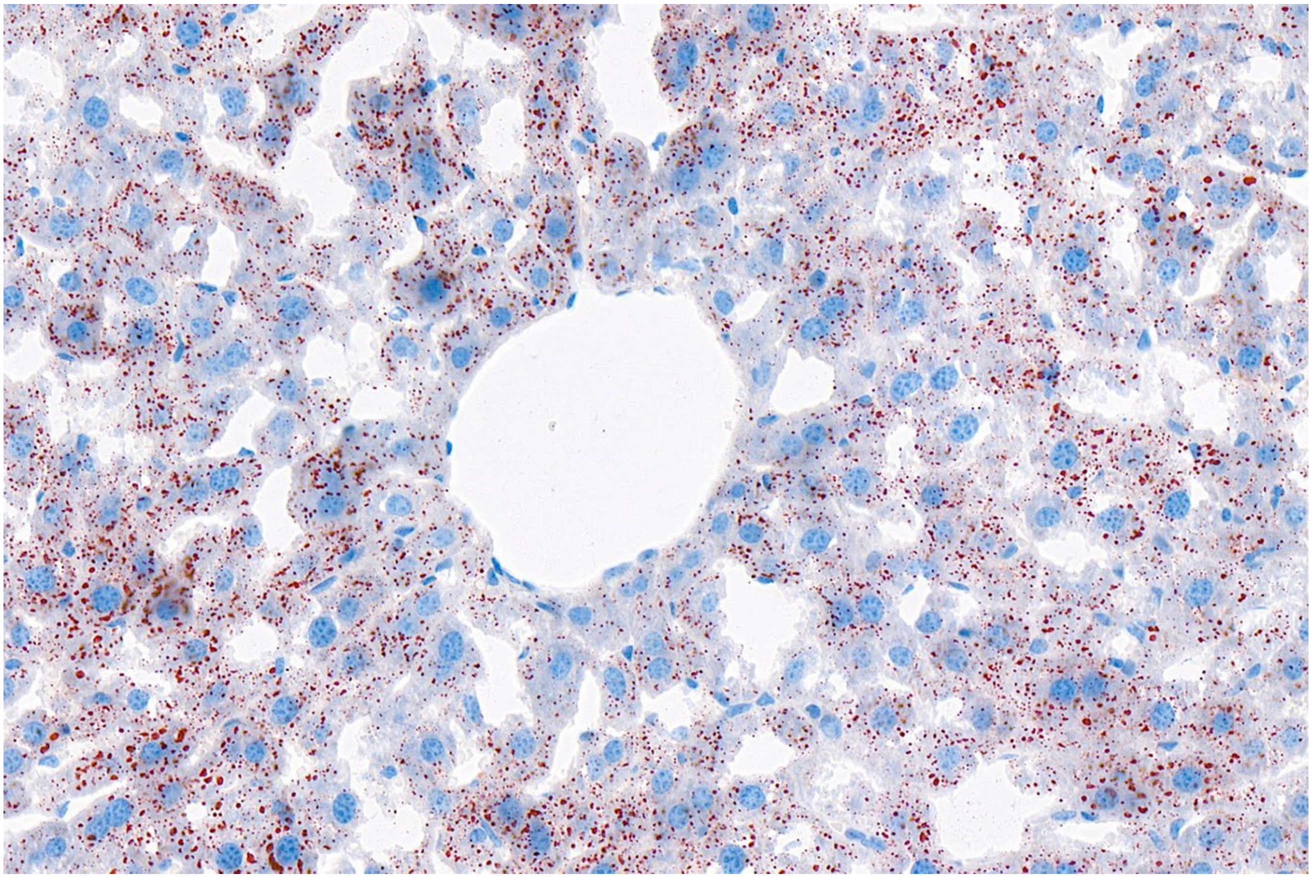

SLBZP Low-dose group

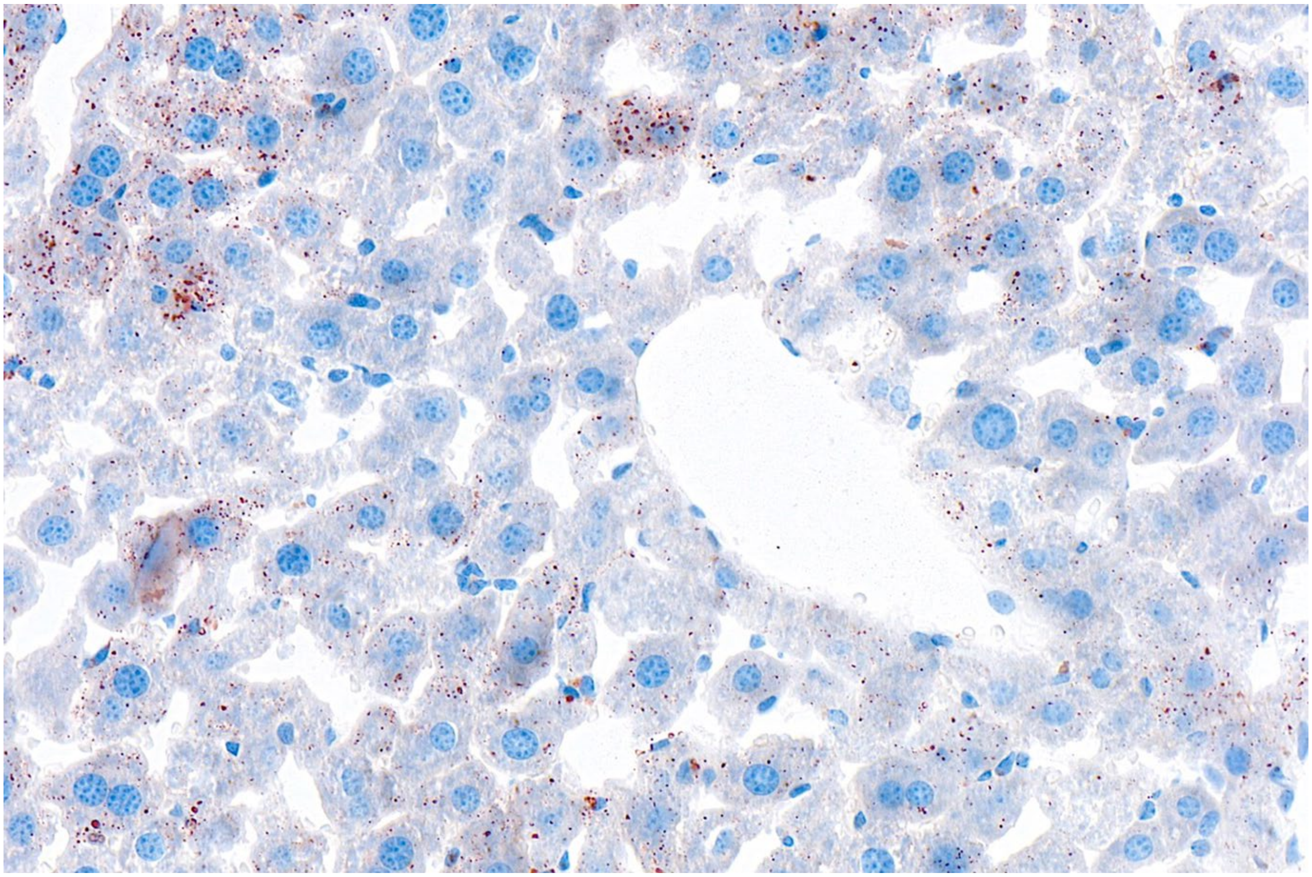

SLBZP Middle-dose group

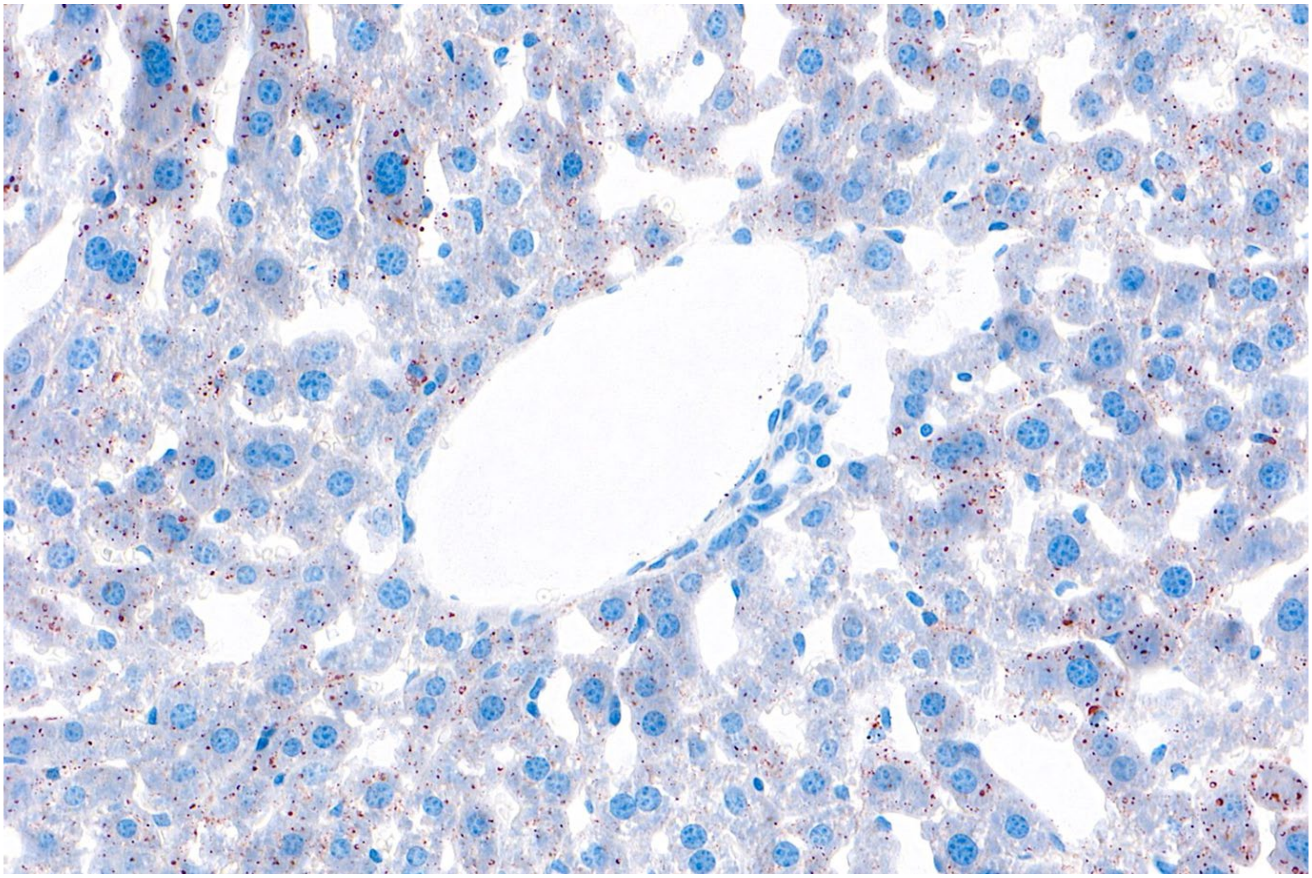

SLBZP High-dose group

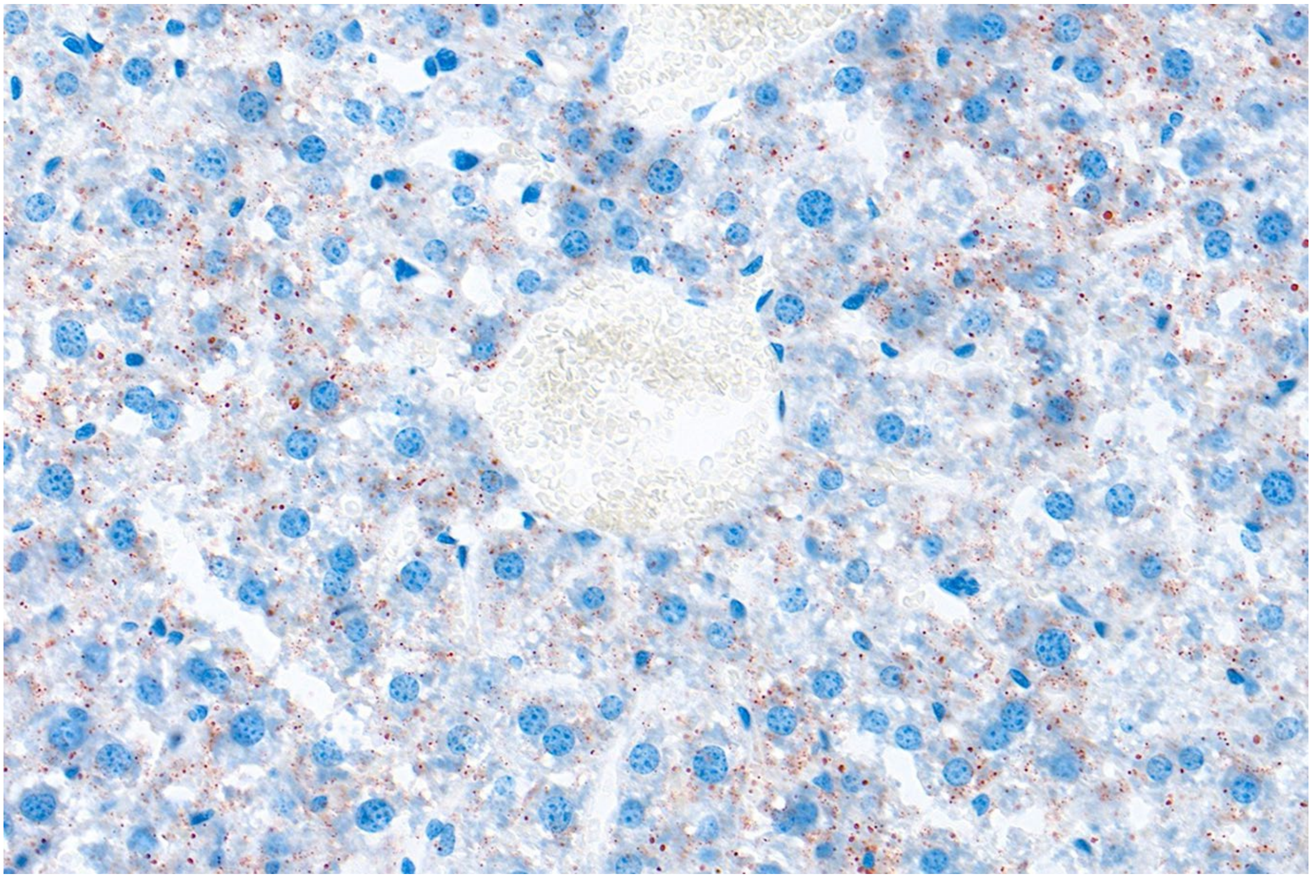

Metformin group

Original bands of protein expression

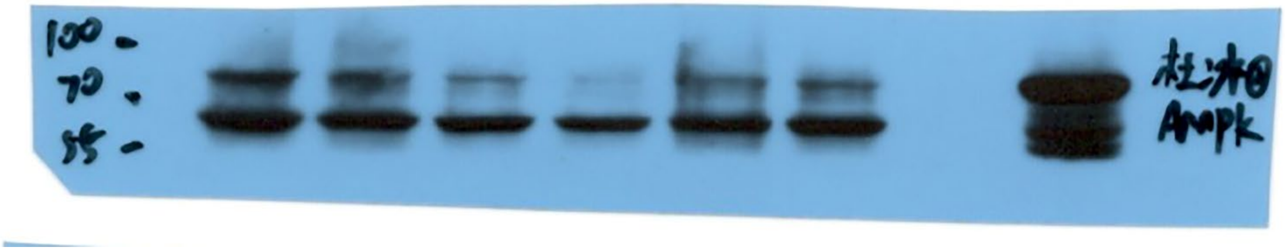

AMPK

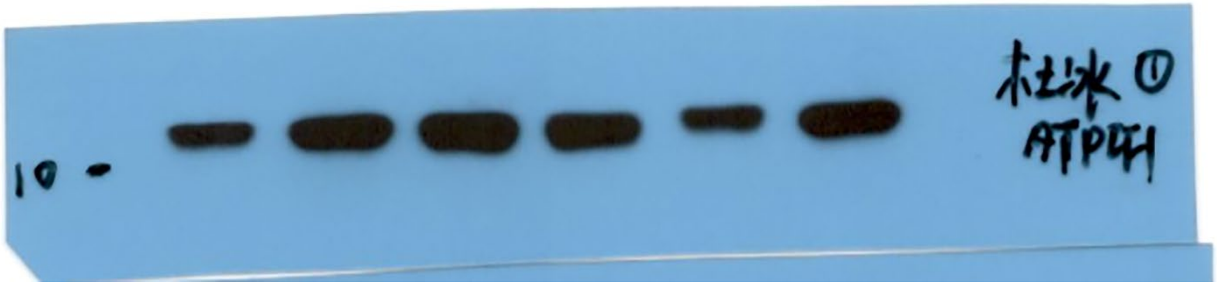

IF1

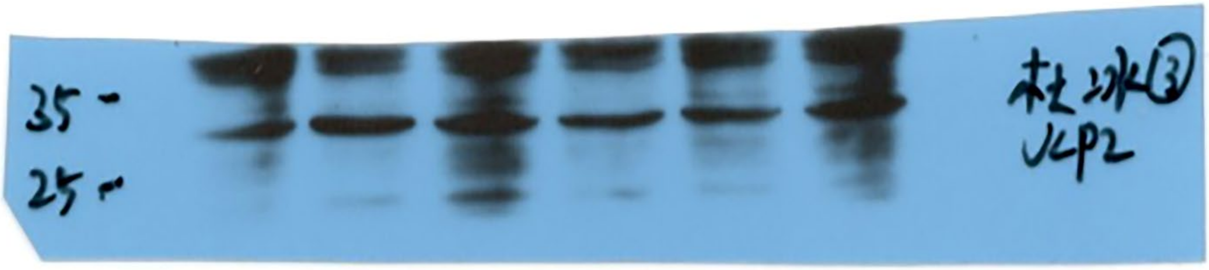

UCP2

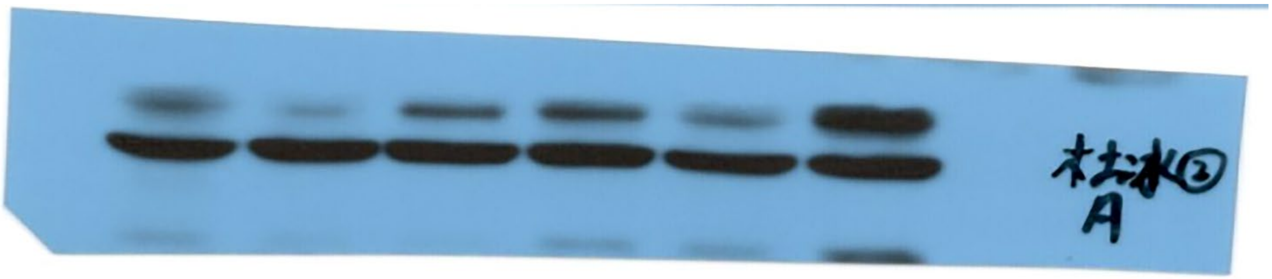

β-actin
